# IRF4 drives clonal evolution and lineage choice in a zebrafish model of T-cell lymphoma

**DOI:** 10.1038/s41467-022-30053-9

**Published:** 2022-05-03

**Authors:** Stella Amanda, Tze King Tan, Jolynn Zu Lin Ong, Madelaine Skolastika Theardy, Regina Wan Ju Wong, Xiao Zi Huang, Muhammad Zulfaqar Ali, Yan Li, Zhiyuan Gong, Hiroshi Inagaki, Ee Yong Foo, Brendan Pang, Soo Yong Tan, Shinsuke Iida, Takaomi Sanda

**Affiliations:** 1 Cancer Science Institute of Singapore, National University of Singapore, 117599 Singapore, Singapore; 2 Department of Biological Sciences, National University of Singapore, 117543 Singapore, Singapore; 3 Department of Pathology and Molecular Diagnostics, Nagoya City University Graduate School of Medical Sciences, Nagoya, 467-8601 Japan; 4 Department of Pathology, Yong Loo Lin School of Medicine, National University of Singapore, 117599 Singapore, Singapore; 5 Department of Hematology and Oncology, Nagoya City University Graduate School of Medical Sciences, Nagoya, Aichi 467-8601 Japan; 6 Department of Medicine, Yong Loo Lin School of Medicine, National University of Singapore, 117599 Singapore, Singapore

**Keywords:** T-cell lymphoma, Cancer models, Oncogenes, Cancer genomics

## Abstract

IRF4 is a master regulator of immunity and is also frequently overexpressed in mature lymphoid neoplasms. Here, we demonstrate the oncogenicity of IRF4 in vivo, its potential effects on T-cell development and clonal evolution using a zebrafish model. IRF4-transgenic zebrafish develop aggressive tumors with massive infiltration of abnormal lymphocytes that spread to distal organs. Many late-stage tumors are mono- or oligoclonal, and tumor cells can expand in recipient animals after transplantation, demonstrating their malignancy. Mutation of *p53* accelerates tumor onset, increases penetrance, and results in tumor heterogeneity. Surprisingly, single-cell RNA-sequencing reveals that the majority of tumor cells are double-negative T-cells, many of which express *tcr-*γ that became dominant as the tumors progress, whereas double-positive T-cells are largely diminished. Gene expression and epigenetic profiling demonstrates that *gata3, mycb, lrrn1, patl1* and *psip1* are specifically activated in tumors, while genes responsible for T-cell differentiation including *id3* are repressed. *IRF4*-driven tumors are sensitive to the BRD inhibitor.

## Abstract (web summary)

IRF4 is a regulator of immune function, and is overexpressed in lymphoid neoplasms. Here, the authors utilise single cell RNA-seq to show the abundance of double-negative T cells in IRF4 driven zebrafish tumour models, and identify sensitivity of these tumours to BRD inhibition.

## Introduction

The transcription factor IRF4, a member of the interferon regulatory factor (IRF) family, is an essential regulator of immunity^1–3^. The expression of IRF4 is highly restricted to hematopoietic and adipocytic lineages, and its level culminates in terminally differentiated lymphocytes, such as plasma cells and activated T-cells^4^. In contrast to other IRF family genes, IRF4 expression is not induced by type I or II interferon but by diverse stimuli, such as T-cell receptor (TCR) signaling in T-cells, IL-4 and CD40 in B-cells, and Toll-like receptor signaling in macrophages^5,6^. IRF4 is required for normal functioning and developmental processes in these cells^1,4,7–11^, particularly as a critical mediator of TCR signaling.

IRF4 has also been implicated as an oncogene in various mature lymphoid neoplasms^12–22^. IRF4 activates oncogenic pathways by transcriptionally regulating downstream target genes, such as MYC, and also coordinately regulate transcriptional program with NF-κB^18,19,22–24^. Functional studies have demonstrated that IRF4 and its binding partners are selective dependency genes; that is, malignant cells are highly dependent on these genes for their proliferation and survival^18,19,23,25^. These in vitro studies suggest that IRF4 can be a driver oncogene; however, the transgenic animal models necessary to investigate the oncogenic property of IRF4 in vivo have not yet been established.

Here, we establish the IRF4-driven lymphoma animal model demonstrating the emergence of highly-invasive tumors that infiltrate multiple organs. Single-cell RNA-sequencing (scRNA-seq) analysis reveal preferential expansion of malignant clones, which can expand in the recipient animals. Gene expression and epigenetic profiling demonstrate that tumors are characterized by the expression of orthologues of human *TCR-*γ, *GATA3*, and *MYC*.

## Results

### Overexpression of wild-type *IRF4* induces aggressive tumors in zebrafish

**Fig. 1:**
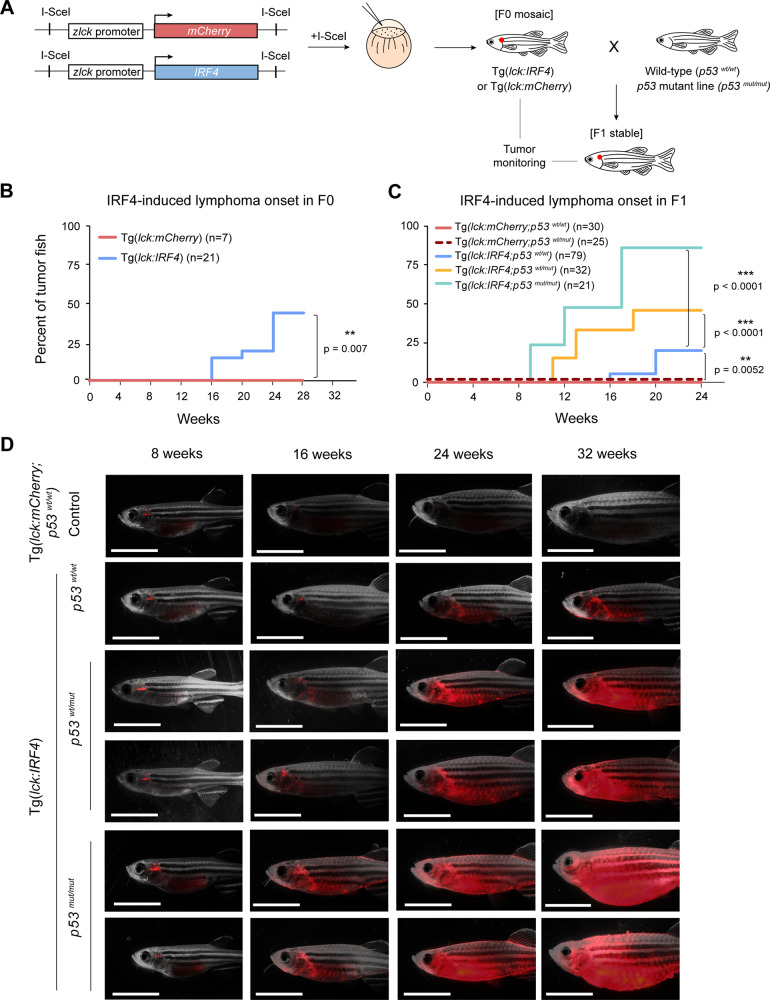
Overexpression of wild-type *IRF4* induces tumors in zebrafish. **A** Schematic diagram of the experimental procedure used to overexpress the human *IRF4* gene under the zebrafish *lck* promoter. **B** Tumor onset in F0 animals was analyzed in the control Tg(*lck:mCherry*) (*n* = 7) and Tg(*lck:IRF4*) transgenic (*n* = 21) groups; *p* = 0.007 according to the Gehan-Breslow-Wilcoxon test. **C** Tumor onset in F1 animals was analyzed in the control Tg(*lck:mCherry;p53*^*wt/wt*^) (*n* = 30), Tg(*lck:mCherry;p53*^*wt/mut*^) (*n* = 25), Tg(*lck:IRF4;p53*^*wt/wt*^) (*n* = 79), Tg(*lck:IRF4;p53*^*wt/mut*^) (*n* = 32) and Tg(*lck:IRF4;p53*^*mut/mut*^) (*n* = 21) groups. *p* = 0.0052 for Tg(*lck:mCherry;p53*^*wt/wt*^) or Tg(*lck:mCherry;p53*^*wt/mut*^) vs Tg(*lck:IRF4;p53*^*wt/wt*^); *p* < 0.0001 for Tg(*lck:IRF4;p53*^*wt/wt*^) vs Tg(*lck:IRF4;p53*^*wt/mut*^); and *p* < 0.0001 for Tg(*lck:IRF4;p53*^*wt/wt*^) vs Tg(*lck:IRF4;p53*^*mut/mut*^) according to the Gehan*-*Breslow-Wilcoxon test. **D** Representative microscopy images of F1 animals: control Tg(*lck:mCherry;p53*^*wt/wt*^), Tg(*lck:IRF4;p53*^*wt/wt*^), Tg(*lck:IRF4;p53*^*wt/mut*^) and Tg(*lck:IRF4;p53*^*mut/mut*^) zebrafish at 8, 16, 24, and 32 weeks postfertilization. Panels show merged fluorescence and brightfield images. Scale bar = 4 mm. Source data are provided as a Source Data file.

We established Tg(*lck:IRF4*) transgenic zebrafish that overexpressed human *IRF4* and a fluorescent marker (mCherry) genes (Fig. 1A). *IRF4* DNA sequence, which includes the IRF superfamily domain and DNA recognition site, is highly conserved among human, mouse and zebrafish (Supplementary Fig. 1A). To analyze the oncogenic ability of *IRF4* in different lineages and stages of lymphocytes, we decided to utilize zebrafish *lck* promoter because *lck* is expressed both in the immature and mature stages of T- and B-lymphocytes in mice (Supplementary Fig. 1B) and zebrafish (Supplementary Fig. 1C), as also reported by others^26–28^. In this setting, tumors arise from the cells where IRF4 expression is oncogenic. To compare the phenotype, we also established the *rag2* promoter-driven *IRF4*-transgenic line because *rag2* expression is more dominantly observed in CD4 + CD8 + double-positive (DP) stage of T-cells (Supplementary Fig. 1B, C). Thus, these systems enable us to detect tissue- and stage-specific oncogenicity of *IRF4*.

After we confirmed the successful integration of the transgenes (*IRF4* and *mCherry*) in somatic tissue of the same animal and actual expression of IRF4 (Supplementary Fig. 1D–G), we monitored the phenotype of transgenic animals. Interestingly, Tg(*lck:IRF4*) zebrafish showed expansion of mCherry signals into the gills (defined as “early-stage”) by 16 weeks in multiple F0 founders (Fig. 1B; Supplementary Fig. 1H) and F1 offspring (Fig. 1C, D). By 32 weeks, the signals had spread throughout the whole body (defined as “late-stage”). In control animals that expressed only the marker gene, Tg(*lck:mCherry*), the mCherry signals disappeared after 16 weeks post-fertilization due to spontaneous regression of the thymus (Fig. 1D; Supplementary Fig. 1H). Because multiple independent F0 animals showed the same phenotype, we concluded that the tumors were induced by *IRF4* overexpression and were not due to the nonspecific effects of random integration of transgenes. Of note, the overall tumor penetrance was lower and tumor onset was later in F1 animals (Fig. 1C) than in F0 animals (Fig. 1B) because F0 fish that developed more aggressive tumors before sex maturation could not be maintained as founder lines. Flow cytometry analysis suggested that the tumor cells were lymphocytes (Supplementary Fig. 1I). In contrast, *rag2* promoter-driven *IRF4* transgenic zebrafish did not cause any tumors (Supplementary Fig. 1J, K).

In human lymphomas, mutation of *p53* gene often coincides with *IRF4* overexpression^29–31^. Thus, we crossed Tg(*lck:IRF4*) with *p53*-mutant fish that expressed the transactivation-dead *p53* variant mutation (*M214K*) similar to that observed in human cancers^32^. Strikingly, 45% of the heterozygous-mutant Tg(*lck:IRF4;p53*^*wt/mut*^) and 85% of the homozygous-mutant Tg(*lck:IRF4;p53*^*mut/mut*^) fish developed tumors by 30 weeks, compared to only 20% in fish with wild-type Tg(*lck:IRF4;p53*^*wt/wt*^) (Fig. 1C, D). Of note, *p53*-mutant animals expressing only *lck-mCherry*, Tg(*lck:mCherry;p53*^*wt/mut*^), did not develop any tumors within 30 weeks (Fig. 1C). Thus, the significant difference in tumor onset and penetrance demonstrated the synergy between the mutation of *p53* and *IRF4* overexpression to induce tumorigenesis. This result also indicated that the presence of one mutant *p53* allele is able to accelerate tumorigenesis, suggesting a potential oncogenic effect of mutant *p53* besides loss of wild-type function, as reported in human cancers^33^.

### *IRF4*-driven zebrafish tumors recapitulate invasive human T-cell lymphoma

**Fig. 2:**
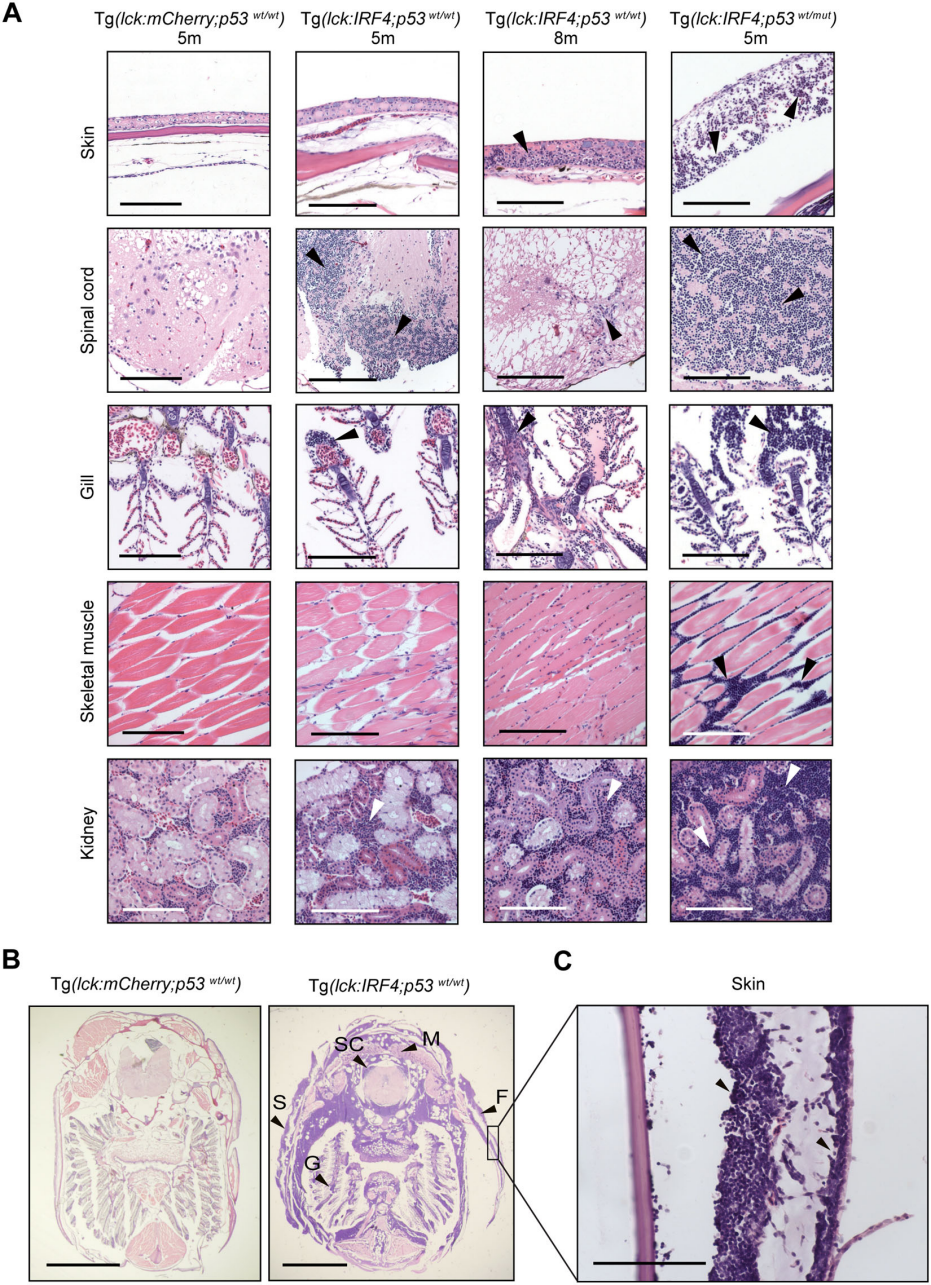
*IRF4*-driven zebrafish tumors recapitulate invasive human T-cell lymphoma. **A** Histopathological examination of H&E-stained tissue sections of representative samples from control Tg(*lck:mCherry;p53*^*wt/wt*^) (*n* = 8), Tg(*lck:IRF4;p53*^*wt/wt*^) (*n* = 8) and Tg(*lck:IRF4;p53*^*wt/mut*^) (*n* = 4) zebrafish at 5 or 8 months. Similar findings were observed in multiple independent animals as shown for each sample. Tumor cells are indicated by white or black arrowheads. Scale bar = 100 μm. **B** Transverse sections of representative samples from Tg(*lck:mCherry;p53*^*wt/wt*^) (*n* = 8) and Tg(*lck:IRF4;p53*^*wt/wt*^) (*n* = 8). F, fin; G, gill; M, muscle; S, skin; and SC, spinal cord. Similar findings were observed in multiple independent animals as shown for each sample. Scale bar = 2 mm. **C** High magnification of skin lesions from Tg(*lck:IRF4;p53*^*wt/wt*^) fish. Scale bar = 100 μm.

We next examined the clinicopathological features of *IRF4*-induced tumors. Histopathological analysis of tumors using whole-body sections from the fish revealed massive infiltration of abnormal cells into the skin, spinal cord, gill, skeletal muscle, and kidney (Fig. 2A), as well as the gastrointestinal tract, testis, and liver (Supplementary Fig. 2A). The mutation of *p53* resulted in more aggressive tumor infiltration into multiple organs at a younger age. In particular, tumor cells infiltrated epidermis, dermis, and subcutaneous tissue (Fig. 2B, C; Supplementary Fig. 2B). These findings recapitulated invasive human T-cell lymphoma, in particular, several types of peripheral T-cell lymphomas in which tumor cells often aggressively infiltrate into the cutaneous region^34–36^.

### Identification of multiple cell populations derived from different stages of lymphocyte differentiation

**Fig. 3:**
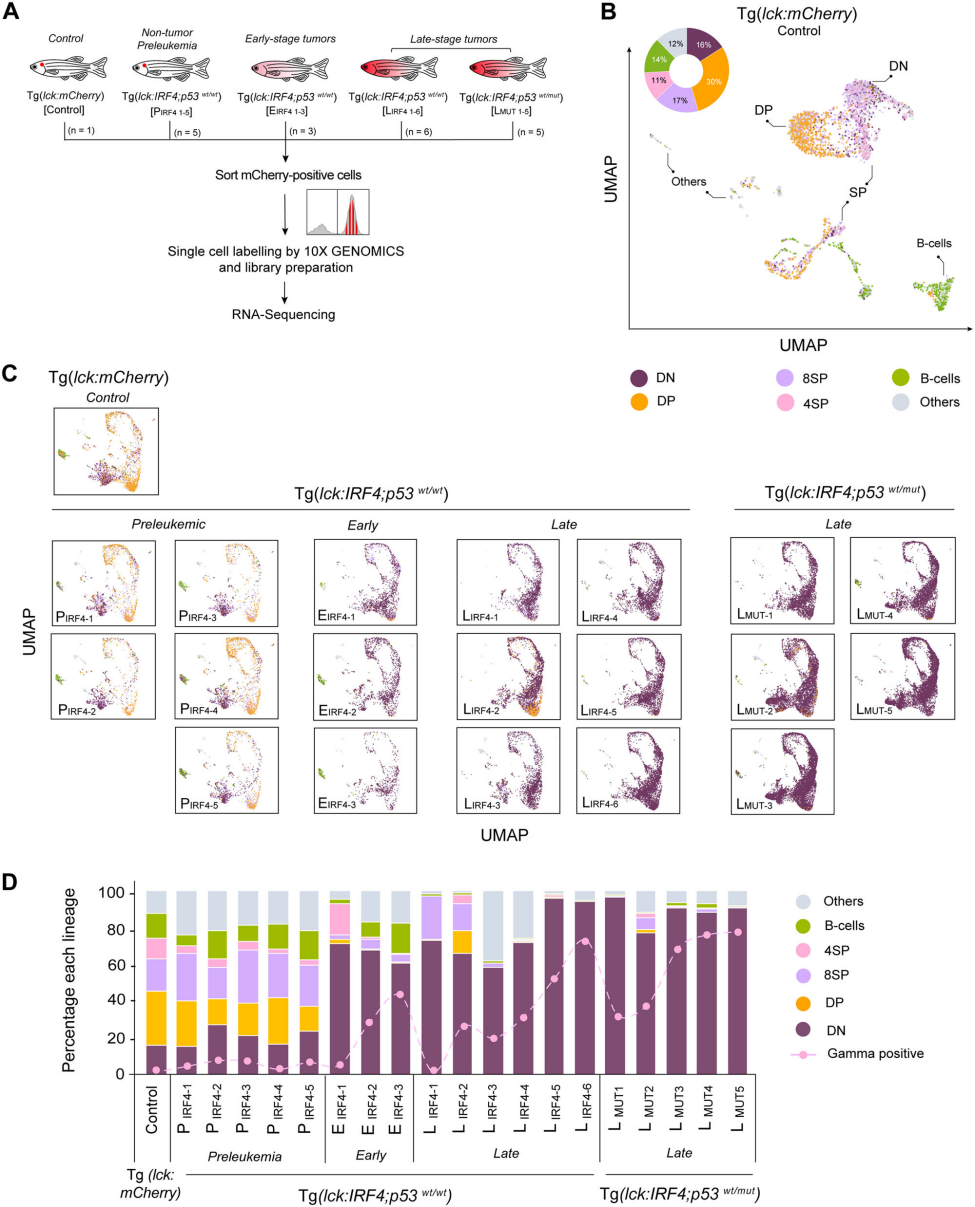
Identification of multiple cell populations derived from different stages of lymphocyte differentiation. **A** Schematic diagram of the sample preparation for scRNA-seq analysis. **B** UMAP plot showing T and B lymphocytes from one control zebrafish. We first selected T and B-cell clusters and then performed a subclustering analysis. Six populations were then defined based on marker gene expression. **C** UMAP plots showing aggregated cells from 20 independent zebrafish. Six populations were annotated using the same criteria used in (**B**). **D** Bar charts showing the percentage of different cell populations in each sample. Source data are provided as a Source Data file.

To determine the specific lineages and stages of tumor cells, we performed scRNA-seq analysis for one normal sample from Tg(*lck:mCherry*) at 3 months old (“control”), five non-tumor preleukemic samples from Tg(*lck:IRF4;p53*^*wt/wt*^) at 3 months old (“P_IRF4_”), three early-stage and six late-stage tumor samples from Tg(*lck:IRF4;p53*^*wt/wt*^) (“E_IRF4_” and “L_IRF4_”), and five late-stage tumor samples from Tg(*lck:IRF4;p53*^*wt/mut*^) fish (“L_MUT_”) (Fig. 3A). We sorted all the mCherry-positive cells obtained from the whole body by flow cytometry and then performed single-cell separation and labeling, followed by library construction and sequencing analysis. After normalization of the mRNA expression values, we selected *mCherry*-positive cells to exclude contaminated cells and then analyzed different cell populations for individual samples or merged data. An average of 5,497 cells were profiled per sample comprising an average of 4,082 UMIs/cell (~3% duplet rate). Zebrafish *lck* gene was expressed in most of the cells (Supplementary Fig. 3A).

As expected, the control sample contained different stages of T-cells, which were classified into: 1) CD4/CD8 double-negative (DN), 2) CD4/CD8 double-positive (DP), 3) CD4 single-positive (4SP), and 4) CD8 single-positive (8SP), based on the expression of *cd4-1*, *cd8a* and other marker genes that have been known to be expressed in zebrafish^37–41^ (Fig. 3B; Supplementary Data 1). The largest population among all *lck-mCherry* positive cells in the control sample was DP T-cells (Fig. 3B). A fraction of DN cells also showed either single expression of *trgc* or *trdc* or co-expression of *trgc* and *trdc*, which are the orthologues of human *TCR-*γ and *TCR-*δ constant regions (Supplementary Fig. 3B), indicating that they are the intermediate stage of DN cells or committed γδ T-cells, respectively, as reported previously in zebrafish^42^. 8SP cells may include intrathymic single-positive (ISP) T-cells, although this population has not been characterized in zebrafish. Together, our results demonstrated that *lck* promoter-driven transgenes are indeed expressed in various stages of normal T-cells and B-cells.

### The expansion of DN and *TCR-*γ-positive T-cells during tumorigenesis

We then used a merged data to visualize the distribution of each cell population at different stages of tumor progression (Fig. 3C). After the sample processing and selection for mCherry-positive cells, we annotated the cell populations using the aforementioned criteria used to analyze the control sample. In marked contrast to the control sample, as the tumor progressed, DN T-cells expanded while DP population was largely diminished (Fig. 3C, D). Surprisingly, within DN T-cells, there was the preferential expansion of the cells expressing *trgc* and majority of these cells were not accompanied with *trdc* expression (Fig. 3D, line; Supplementary Fig. 3C), suggesting that they may be the intermediate stages of DN T-cells or aberrant *trgc*-expressing T-cells. Additionally, several genes known to be expressed in the γδ T-cells, such as *sox4a* and one of *granzymes* (*gzmk*)^34,43–46^, were highly expressed in tumor samples (Supplementary Fig. 3D). Those *trgc*-positive cells further expanded in many tumor samples at the late stage while other populations of γδ T-cells stayed throughout tumor progression (Supplementary Fig. 3E). They accounted for approximately 75% of the total T-cells, which was a marked contrast to the percentage in control or pre-leukemic samples (2–10%) (Fig. 3D). We validated this result using independent qRT-PCR analysis of 20 additional tumor samples, which confirmed the upregulation of *trgc* expression in all tumor samples compared to that in control (Supplementary Fig. 3F). It is noteworthy that *trgc* is not expressed in the existing zebrafish T-cell acute lymphoblastic leukemia (T-ALL) model driven by *rag2-myc*^47^ (Supplementary Fig. 3G). Hence, the expansion of *TCR-*γ*-*positive CD4-/CD8-DN T-cells is unique to and is a common phenotype in our zebrafish model.

### IRF4-driven tumor cells possess malignant features

**Fig. 4:**
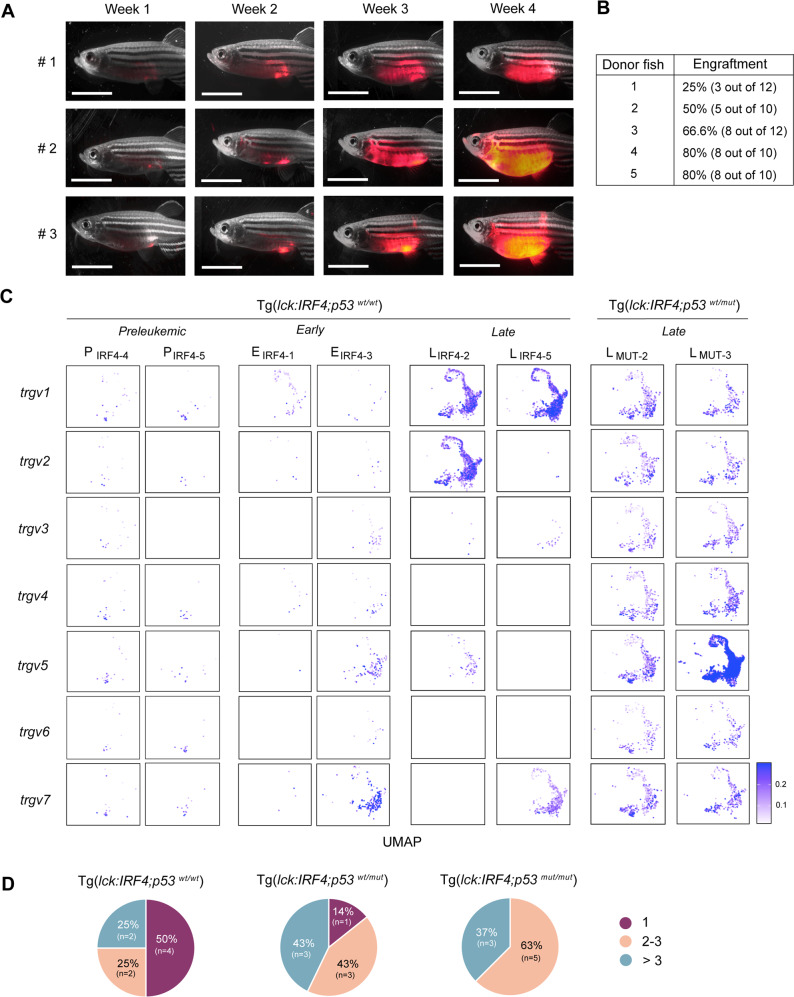
IRF4-driven tumor cells possess malignant features. **A** Representative microscopy images of three recipient fish at 1, 2, 3, and 4 weeks after the first transplantation. Panels show merged fluorescence and brightfield images. Scale bar = 4 mm. **B** Table showing the engraftment rate of five independent donor tumor fish. **C** Gene expression pattern of the *tcr-*γ variable regions in eight representative samples. The cells in the UMAP plot are colored by the marker gene expression level. **D** Pie charts showing the percentage of mono-, oligo-, and polyclonal tumors analyzed by qRT-PCR in three different genotype settings. Source data are provided as a Source Data file.

We next examined if the proliferating cells are indeed malignant cells. We transplanted FACS-sorted mCherry-positive cells from Tg(*lck:IRF4;p53*^*wt/wt*^) fish into immunocompromised zebrafish (*rag2*^*E450fs*^-mutant strain) and monitored the engraftment and expansion of mCherry-positive cells. Representative images from three independent experiments with different donor fish are shown (Fig. 4A). This analysis demonstrated that the donor cells from different tumors were able to be engrafted at a high frequency (Fig. 4B) and expanded to the whole body within a month. A serial transplantation assay further revealed that these cells could expand and survive for a long time (Supplementary Fig. 4A) and many tumors were able to be maintained over 1 year. Histopathological examinations revealed that the cells primarily infiltrated the skin, muscle, kidney, liver and spinal cord, which was similar to the observations in the original tumors, except the thymus and gills (Supplementary Fig. 4B). These results indicated that the transplanted cells contained self-renewing cells, which can generate tumor cells for a long term.

Furthermore, scRNA-seq analysis showed that the T-cell population in the preleukemic and early-stage tumors expressed multiple *trgv* genes (Fig. 4C, left), which are the variable genes of zebrafish *TCR-*γ gene locus (Supplementary Fig. 4C, D), while those in the late-stage samples expressed only one or a few *trgv* genes at high level (middle), indicating that the tumors were polyclonal at the early stage and became oligo- or monoclonal at the late stage. In the *p53*-mutant background, expression of multiple *trgv* genes was observed, even at the late stage (right). To validate this finding, we performed qRT-PCR for transcripts between *TCR-*γ constant and different variable regions from independent tumor samples (Supplementary Fig. 4E). This analysis revealed that among eight Tg(*lck:IRF4;p53*^*wt/wt*^) tumor samples that expressed the *trgc* gene, 50% (4 of 8) of them expressed only one *trgv* transcript (Fig. 4D). In contrast, the majority of tumors expressed multiple *trgv* transcripts in the *p53*-mutant background. This result further suggests that the mutation of *p53* confers a strong proliferative or survival advantage to multiple different clones at the early stage of tumorigenesis, thereby contributing to tumor heterogeneity.

### IRF4-driven tumors are characterized by high expression of *mycb* and *gata3*

**Fig. 5:**
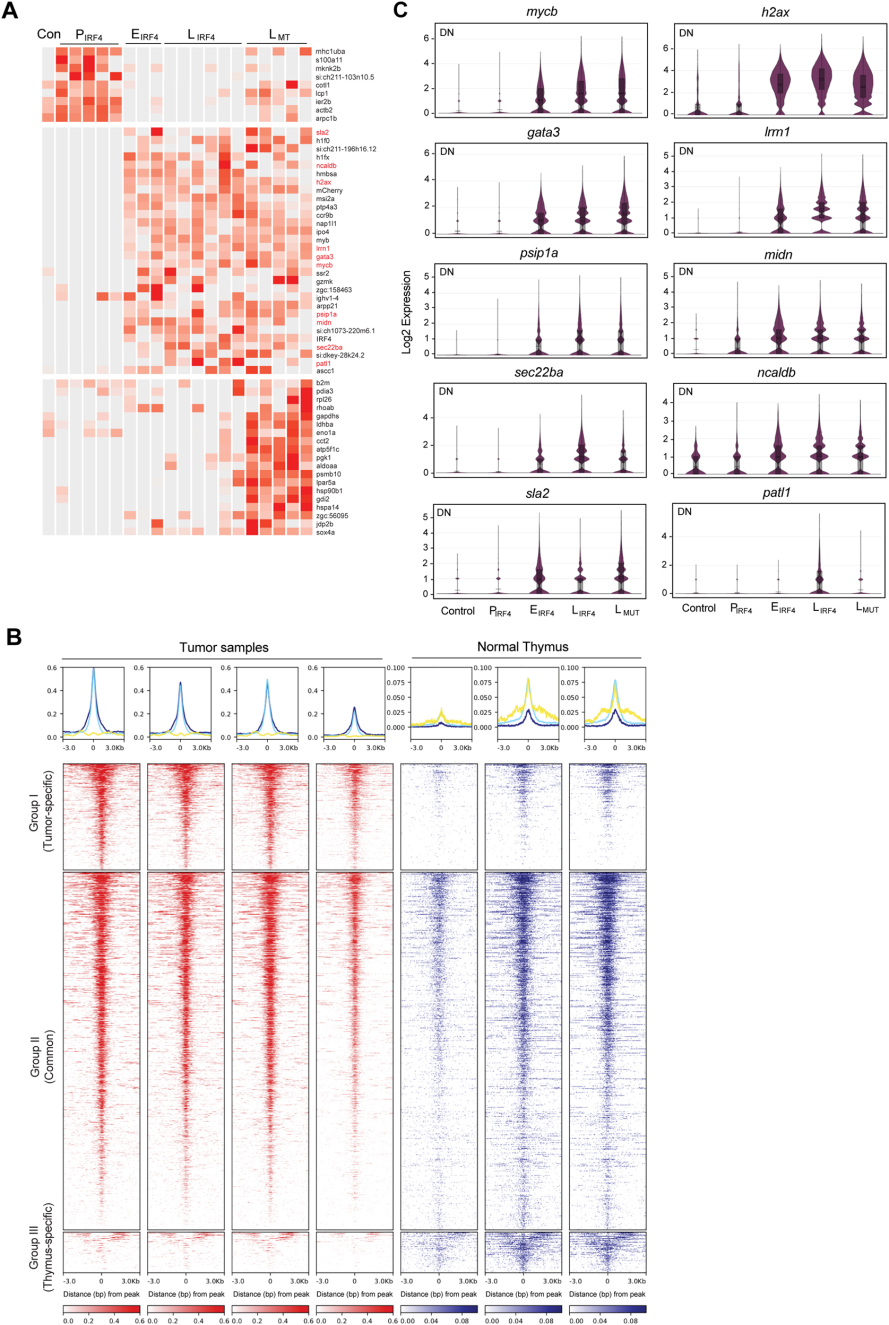
IRF4-driven tumors are characterized by high expression of *mycb* and *gata3*. **A** Heatmap showing the significantly upregulated genes from one-to-one differential gene analysis of scRNA-seq data between DN populations of different tumor stages. **B** Density plots (bottom) show the distribution of H3K27ac signals in tumor and normal thymus samples. All significant peaks (FDR < 0.05) were first detected and then classified into three classes of regions (Groups I-III). The color scale represents the intensity of signals and distances (3,000 bps) from the center (0) of peaks. Metagene plots (top) show the distribution of H3K27ac signals from the center for Group I (log2FC ≥ 1) (blue), Group II (−1 < log2FC < 1) (light blue) and Group III (log2FC ≤ −1) (yellow) regions. **C** Violin plots showing the expression of ten genes in DN cell populations across tumor stages analyzed by scRNA-seq (total cell number after merging of biologically independent samples: Control *n* = 557 cells; P_IRF4_ *n* = 2,222 cells; E_IRF4_ *n* = 3,365; L_IRF4_ *n* = 15,280 cells; L_MT_ *n* = 28,205 cells). Black vertical line indicates max and min values. Box indicates the third and first quartiles. Horizontal lines in the box indicate mean and median values. Source data are provided as a Source Data file.

We next aimed to elucidate potential mechanisms by which IRF4 induces tumorigenesis. We performed one-to-one differential gene expression analysis using scRNA-seq data between DN populations of different tumor stages, and selected genes, which were significantly upregulated (*p* value <0.05) (Fig. 5A). For example, before the lymphoma phenotype was evident in the preleukemic stage, one of the earliest genes to be upregulated was *s100a11* (Fig. 5A, top), which overexpression has been reported to promote cell proliferation, antiapoptotic, and tumor metastasis in various cancers^48,49^. Interestingly, several genes that have been known to be involved in T-cell tumorigenesis, including *mycb* and *gata3*, the orthologues of human *MYC* and *GATA3*, were specifically upregulated in tumor samples (Fig. 5A, middle). On the other hand, multiple metabolic enzymes (e.g., *aldoaa*) were significantly upregulated in late-stage tumors with the *p53* mutation (Fig. 5A, bottom).

To further analyze molecular mechanism, we also performed a differential epigenetic profiling by chromatin-immunoprecipitation-sequencing (ChIP-seq) analysis for an active histone mark (H3K27ac) for multiple bulk tumor and normal thymus samples and performed a differential binding analysis (Supplementary Data 2). Density plots and metagene plots (Fig. 5B) represent three groups of regions: i) specifically activated in tumors (Group I), ii) commonly activated both in tumors and normal thymus (Group II), and iii) specifically activated in the normal thymus (Group III), based on statistical analysis (FDR < 5% & log2 fold-change >1). Regions activated in tumors (Group I + II) were enriched for DNA binding motifs recognized by IRF family transcription factors (Supplementary Fig. 5A). In fact, Group I included *mycb* and *gata3*, which were associated with high level of H3K27ac signals in multiple tumor samples but not in the normal thymus (Supplementary Fig. 5B). We also identified super-enhancers, clusters of active enhancers, which are often present at genes that characterize cell type^50,51^. This analysis demonstrated that many genes involved in T-cell development, including *bcl11ba, ikzf1, lck, runx3* and *tcf7*, were associated with super-enhancers in multiple tumors and normal thymus samples (Supplementary Fig. 5C and Supplementary Data 3). Notably, super-enhancers at *mycb/fam49ba, gata3* and PI3K kinase subunit genes *(pik3cd* and *pik3c2b*) were found to be more frequent in tumors (≥3 out of 4 tumors, and 1≤ out of 3 thymus samples), suggesting their requirement in tumorigenesis (Supplementary Fig. 5D). We performed independent validation by qRT-PCR of 20 additional samples, which demonstrated the upregulation of *mycb* and *gata3* (Supplementary Fig. 5E).

Notably, besides *mycb* and *gata3*, eight genes (*h2afx, lrrn1, midn, ncaldb, patl1, psip1a, sec22ba*, and *sla2*) which were not well studied in cancers, were commonly selected by gene expression and differential epigenetic analyses (Fig. 5A, middle; Fig. 5C; Supplementary Fig. 5B). In particular, expression of *lrrn1, patl1* and *psip1a* were highly specific to tumor cells; 0% of normal thymus and less than 3% preleukemic samples were positive for all three genes, while up to 46% of tumor samples were positive (Supplementary Fig. 5F). The orthologues of five genes, *LRRN1, PSIP1, PATL, H2AFX*, and *MIDN*, were significantly upregulated in human γδ T-cell lymphoma (GD-TCL) samples as compared to normal γδ T-cells (Supplementary Fig. 5G)^52^, suggesting their involvement in T-cell lymphomagenesis.

### Downregulation of p53 targets and upregulation of anti-apoptotic genes

**Fig. 6:**
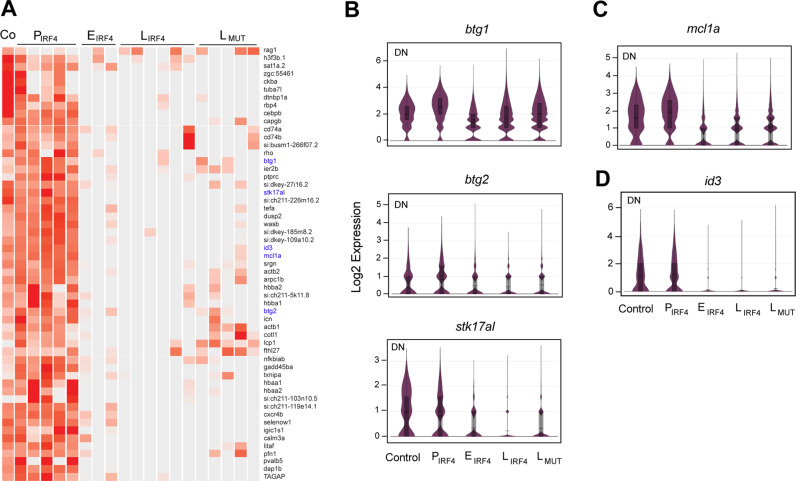
Downregulation of p53 targets and *id3*. **A** Heatmap showing the significantly downregulated genes from one-to-one differential gene analysis of scRNA-seq data between DN populations of different tumor stages. **B**–**D** Violin plots showing the expression of *btg1, btg2* and *stk17al* (**B**)*, mcl1a* (**C**), *id3* (**D**) in DN cell populations across tumor stages analyzed by scRNA-seq (total cell number after merging of biologically independent samples: Control *n* = 557 cells; P_IRF4_ *n* = 2,222 cells; E_IRF4_ *n* = 3,365; L_IRF4_ *n* = 15,280 cells; L_MT_ *n* = 28,205 cells). See Fig. 5C legend for the details of violin plots. Source data are provided as a Source Data file.

We next focused on genes which were significantly downregulated (*p*-value <0.05) in the DN population of tumor samples (Fig. 6A). This list included genes associated with tumor suppressor and apoptotic pathways. Several genes known to be downstream of p53 pathway, namely *btg1, btg2*, and *stk17al*^53–55^, were downregulated to an indistinguishable level with that in tumor cells with *p53* mutation (Fig. 6A, top; Fig. 6B), suggesting that p53 activity might be lost during tumorigenesis even in the wild-type setting. However, *p53* gene was still expressed and also not mutated in these samples based on PCR and sequencing analysis, indicating that p53 might be functionally suppressed via a different mechanism. It is also noteworthy that one of antiapoptotic BH3 family genes, *mcl1a*, was also downregulated in early-stage tumors although its expression slightly recovered in late-stage of tumors (Fig. 6C). This suggested that pro-apoptotic signal might be dominant and thus the tumor cells underwent apoptosis at the early stage while they became resistant to apoptosis at the late stage.

### IRF4 overexpression affects cell differentiation and lineage choice

Interestingly, one of the most significantly downregulated genes in DN cells was *id3*, which was expressed in preleukemic samples but almost completely suppressed in tumor samples (Fig. 6A, top; Fig. 6D). This gene was also found in Group III by ChIP-seq analysis (Fig. 5B; Supplementary Fig. 6A). Previous reports showed that conditional knockout of *id3* in mice under *Lck-Cre* impaired γδ T-cell development and led to the expansion of the immature stage of γδ T-cells^56,57^, similar to the phenotype observed in our zebrafish model. Additionally, we performed one-to-one differential gene analysis of the DP, 4SP, and 8SP between each tumor stage. This analysis revealed that although these populations do not undergo abnormal expansion like DN cells, their expression profiles still deviated from the normal pattern. For example, the expression level of *cd4-1, cd8a/b, rorc*, and *rag1*, which are normally upregulated in the DP stage^58^, was lower in the tumor samples (Supplementary Fig. 6B). Of note, a forced overexpression of IRF4 in an IRF4-negative human T-ALL cell line (HPB-ALL), which exhibits DP stage immunophenotype, significantly inhibited cell growth and induced apoptosis (Supplementary Fig. 6C, D), suggesting that IRF4 overexpression at this stage of thymocytes is potentially lethal and thus IRF4-positive DP cells cannot expand. These results indicated that although cells are differentiating in the αβ-lineage continuum, *IRF4* overexpression interfered with the differentiation process.

### IRF4-driven T-cell tumors are sensitive to a small-molecule BRD inhibitor

**Fig. 7:**
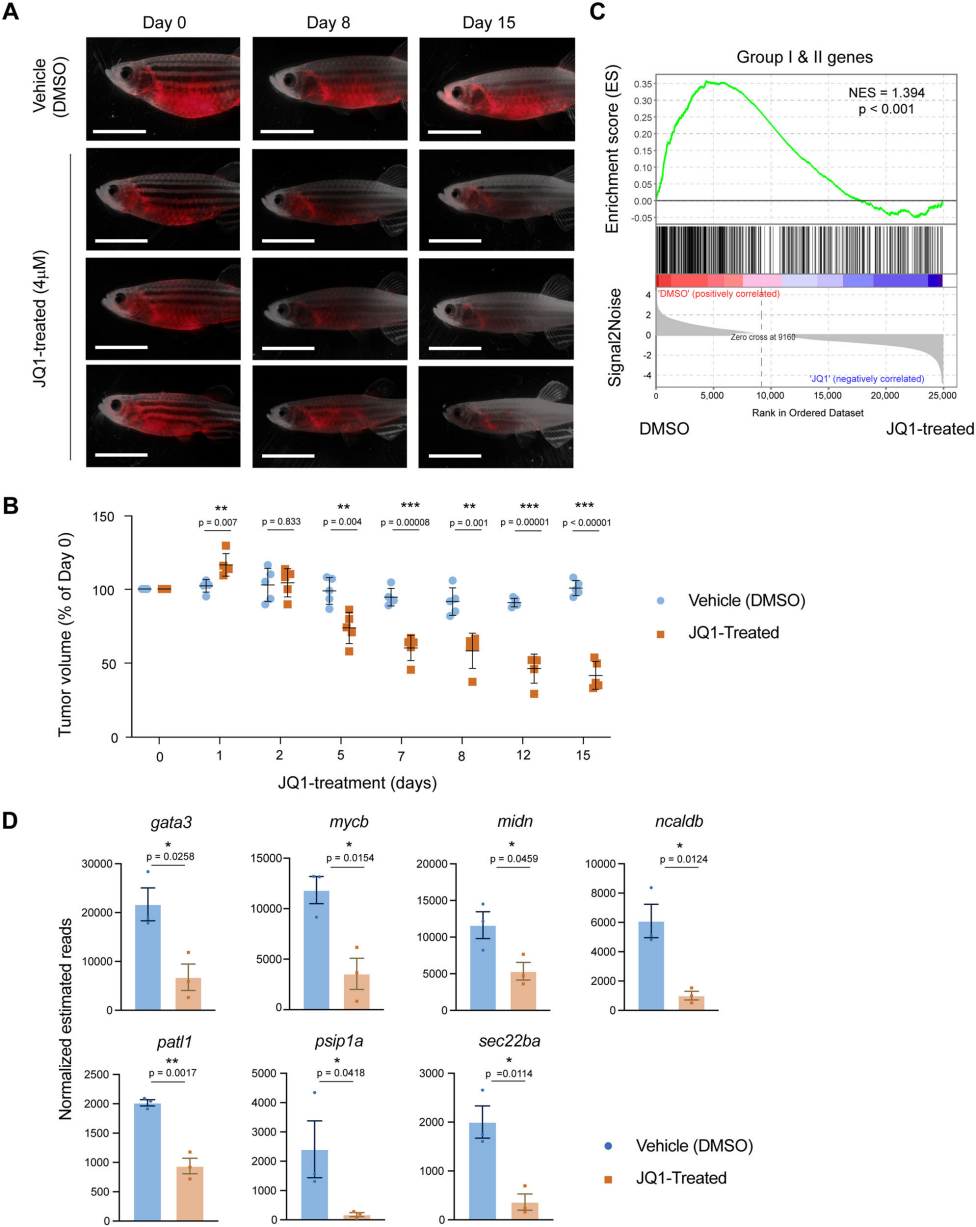
*IRF4*-driven T-cell tumors are sensitive to a small-molecule BRD inhibitor. **A** Representative microscopy images of Tg*(lck:IRF4;p53*^*wt/wt*^*)* transgenic fish treated with vehicle (DMSO; *n* = 5) or JQ1 (4 μM; *n* = 5). Panels show merged fluorescence and brightfield images. Scale bar = 4 mm. **B** Quantification of tumor volume for five independent animals on days 1, 2, 5, 7, 8, 12 and 15 compared to that on day 0. Integrated fluorescence intensity was quantified by ImageJ (NIH) software and normalized to body size; *p < 0.05, **p < 0.01, and ***p < 0.001 by two-tailed unpaired Student’s *t*-tests. Bars indicate mean and SD values. Day 1 *p* = 0.07; day 2 *p* = 0.83; day 5 *p* = 0.04; day 7 *p* = 7.7 × 10^−5^; day 8 *p* = 0.001; day 12 *p* = 1 × 10^−5^; day 15 *p* = 1.7 × 10^−5^. **C** Gene set enrichment analysis plots showing the overall pattern of gene expression changes of Group I + II genes upon JQ1 treatment. Each solid bar represents one gene within the gene set. The normalized enrichment scores (NES) by dataset permutations and *p*-values are indicated. **D** Bar charts showing the expression level of seven selected genes in control and JQ1-treated samples (*n* = 3 independent animals for each group). The data represent the mean ± SEM (shown as bars) of replicate samples; *p < 0.05 and **p < 0.01 by two-tailed unpaired Student’s *t*-tests. Source data are provided as a Source Data file.

Lastly, we tested a potential therapeutic option in our zebrafish lymphoma model. Gene expression analysis indicated that *mycb* was predominantly upregulated in IRF4-driven T-cell tumors. Recently, accumulating studies show that *MYC*-overexpressing cancers are sensitive to small-molecule BRD inhibitors, such as JQ1^59–61^. BRD proteins interact with acetylated histones and promote transcriptional initiation and elongation processes. Genes highly activated in tumor cells are known to be strongly inhibited by JQ1 in various cancers^60–64^. Strikingly, the fish tumors showed a remarkable response after JQ1 treatment (Fig. 7A). The tumor volumes were significantly reduced compared to those of the control fish treated with DMSO (Fig. 7B). To further confirm if genes activated in tumors are sensitive to JQ1, we performed an RNA-seq analysis for three JQ1-treated and three DMSO-treated samples at Day3 (Supplementary Data 4). As expected, many of Groups I and II genes that represent genes highly activated in tumor cells (from Fig. 5B) were downregulated by JQ1 treatment, as demonstrated by the gene set enrichment analysis (Fig. 7C). In fact, seven of 10 shortlisted genes (from Fig. 5A, red) were significantly downregulated after the treatment (Fig. 7D), further suggesting that their expressions are associated with tumor maintenance. Together, our data indicated that the IRF4-driven tumors depended on the transcriptional activity and also demonstrated the applicability of our transgenic zebrafish model for drug testing.

## Discussion

IRF4 has long been implicated as an oncogene in lymphoid malignancies. Since this gene was cloned at the breakpoint of a chromosomal translocation, t(6;14)(p25;q32), involving the *IgH* gene locus in MM cells^65^, a plethora of in vitro and clinical studies have reported the abnormal overexpression and oncogenic roles of IRF4 in various mature lymphoid neoplasms^13–16,18–22,25^. IRF4 directly induces *MYC* expression and also coordinately regulate the transcription program with NF-kB pathway^19,22^. However, the oncogenicity of *IRF4* in vivo has not been reported.

Here, we demonstrated that overexpression of wild-type IRF4 can cause the development of aggressive T-cell lymphoma. Although our model resembled human GD-TCL based on TCR-γ expression, it also possesses several common features that are generally observed in mature T-cell neoplasms, such as high level of expression of *GATA3* and *MYC*, and the infiltration of tumor cells into the surrounding tissues and distal organs, in particular, cutaneous regions. This finding was different from the phenotype reported for the *rag2-Myc*-induced zebrafish T-ALL model, in which tumor cells typically spread through the vessels and showed the expansion of DP T-cells^66–68^. Of note, a previous study reported that overexpression of IRF4 under the *E*μ promoter did not cause tumors in mice^69^. Similarly, the *rag2*-promoter-driven *IRF4*-transgenic zebrafish did not cause any tumors in our study. This discrepancy is likely due to the different promoter systems that can be activated in different stages and linages of lymphocytes. In general, *rag2* promoter is more active in the DP stage of T-cells and our study also showed that *IRF4* overexpression results in downregulation of *rag1/2* expression. The *E*μ promoter is more active in immature B-cells, as shown in a *MYC*-induced lymphoma model using the same promoter that predominantly developed pre-B lymphomas and mixed pre-B and B-cell lymphomas^70^. In contrast, the zebrafish *lck* promoter is active both in immature precursor lymphocytes and mature T-cells. LCK is also an immediate downstream target of the TCR, while IRF4 mediates the T-cell activation process through TCR signaling^7,9–11^. Thus, there is a positive feed-forward loop between IRF4 and TCR-LCK. Additionally, the kinetics and expression pattern of *IRF4* expression are positively correlated with the strength of TCR signaling, which in turn, affects normal T-cell population expansion^10,11,71^. Therefore, a specific cellular context that possesses active TCR signaling including LCK expression may be required for the full oncogenic ability of IRF4 in vivo.

However, the expansion of DN T-cells, many of which expressed *TCR-*γ, was initially unexpected. Our analysis suggested that tumor cells are likely derived from the intermediate stage of DN T-cells that can generate both γδ T-cells and αβ T-cells. Although this phenotype may be specific to the experimental setting, our study provides an important implication: overexpression of IRF4 in the DN stage affects cell differentiation and lineage choice. A potential reason for this outcome can be explained by several consequential factors. First, our and other studies indicated that IRF4 transcriptionally upregulates several molecules downstream of the TCR signaling pathway so that the cells can respond strongly upon TCR activation. It has been known that the lineage fate of T-cells (αβ vs γδ) can be influenced by the strength of TCR signaling at DN stage, in which stronger signal favors the development of the γδ lineage^72,73^. Thus, overexpression of IRF4 prior to this stage might affect the lineage choice. Similarly, several genes responsible for T-cell differentiation such as *rag1* and *id3* were downregulated in DN stage of tumor samples. Thus, IRF4-overexpressing cells may not be able to further rearrange TCR-α or β and instead be committed to γδ lineage. Second, the DP stage of T-cells normally undergo positive and negative selections, by which the cells that lack or have excess activity of TCR signaling undergo apoptosis. In a physiological condition, IRF4 expression is strictly restricted to mature T-cells, which have successfully passed the DP checkpoint. Hence, ectopic overexpression of IRF4 in DP stage may cause excess TCR signals, which potentially triggers apoptosis at this stage of thymocytes development. Therefore, IRF4 overexpression at this stage could be lethal. The fact that *rag2* promoter-driven system does not cause tumors, but *rag2-myc* does also supports this possibility. This finding also supports the fact that IRF4 has not been implicated as an oncogene in T-ALL, which mainly proliferate at late DN and DP stage of T-cells. In fact, overexpression of IRF4 in DP stage T-ALL cell lines caused apoptosis. Similar finding has been reported in B-cell acute lymphoblastic leukemia and chronic lymphocytic leukemia cases, which implicated a tumor-suppressive role of IRF4^23,74–76^. Lastly, γδ T-cells are relatively more frequent in amphibians, reptiles or teleost fish, including zebrafish; and the intermediate stage of DN T-cells express TCR-γ alone as previously reported^42^. Thus, high activation of TCR-γ-mediated signaling at the intermediate stage of DN T-cells might result in the preferential expansion of this type of T-cells.

Additionally, we observed that IRF4-driven tumor cells exhibited several important malignant features. Pre- or malignant cells undergo clonal competition during tumorigenesis, which ultimately leads to mono- or oligoclonal proliferation of T-cells. These cells were able to be engrafted and survive for a long time in the recipient animals, suggesting the existence of a leukemia-initiating cell population. Furthermore, the mutation of *p53* confers an additional advantage, by which the cells can resist apoptotic cell death and thus collaborate with IRF4 overexpression. In this setting, the clones may bypass the severe competition and thus exhibit tumor heterogeneity. Our study also demonstrated that heterozygous mutation of *p53* in the transactivation domain is able to accelerate tumorigenesis. It has been reported that the mutant p53 functions as a gain-of-function by interacting with other transcription factors, besides the loss of wild-type protein^33,77^. We would like to investigate these aspects in a future study.

In conclusion, we established an *IRF4*-driven zebrafish lymphoma model. Our results with a small-molecule BRD inhibitor also demonstrated the feasibility of this model for drug testing. Thus, faced with the pressing need to understand the pathogenesis and find effective therapies for T-cell lymphoma, our work provides the necessary animal model system, which would be a useful platform for further mechanistic studies as well as for testing therapeutic options to improve the treatment of this disease.

## Methods

### Zebrafish studies

All zebrafish protocols (BR17-0351, R17-0353, BR18-1349, R18-1350, BR18-0526, R19-1182 BR21-0082, R21-0084) were approved by the Institutional Animal Care and Use Committee of the National University of Singapore and were performed according to their recommendations. The zebrafish *lck* promoter construct was kindly provided by Dr. Hui Feng (Boston University, Boston). Animals used in this study were of *Danio rerio* species with the specific strains explained, as follows. The *p53*-mutant zebrafish line (*M214K* mutant) was kindly provided by Dr. Thomas Look (Dana-Farber Cancer Institute, Boston)^28^. An immunocompromised strain (*rag2*^*E450fs*^ mutant) was established and kindly provided by Dr. David Langenau’s laboratory^78^. We established other transgenic animals using the Singapore wild-type strain, as follows. The human *IRF4* or *mCherry* gene was cloned downstream of the *lck* or *rag2* promoter into a backbone vector. The plasmids were resuspended in buffer and linearized using the I-SceI enzyme (New England Biolabs). The solution was injected into one-cell-stage zebrafish embryos to establish the Tg(*lck:IRF4*) line. Tg(*lck:mCherry*) animals injected with only *lck-mCherry* were established as a control line. Tg(*rag2-IRF4*) was also established as comparison for Tg(*lck:IRF4*).

### Tumor monitoring

For the tumor monitoring study, brightfield and fluorescent images were captured using a Nikon Stereomicroscope SMZ800N and DS-Ri2 camera. Image acquisition was performed using NIS-Elements D software. Zebrafish of mixed gender were monitored from 2 weeks of age weekly for 1 year or until the animals died whichever was earlier. Tumor monitoring was performed in F0 animals, control Tg(*lck:mCherry*) (*n* = 7) and Tg(*lck:IRF4*) transgenic (*n* = 21). Tumor monitoring was performed in F1 animals, control Tg(*lck:mCherry;p53*^*wt/wt*^) (*n* = 30), Tg(*lck:mCherry;p53*^*wt/mut*^) (*n* = 25), Tg(*lck:IRF4;p53*^*wt/wt*^) (*n* = 79), Tg(*lck:IRF4;p53*^*wt/mut*^) (*n* = 32) and Tg(*lck:IRF4;p53*^*mut/mut*^) (*n* = 21), and Tg(*rag2:IRF4*) (*n* = 13). Early-stage tumors were defined by the expansion of mCherry-positive cells outside the thymus, including in the anterior musculature, orbit, nasal placode, gills, and pectoral fins. Tumors were considered late-stage when mCherry-positive cells had spread to more than 50% of the zebrafish body.

### Drug treatment

The small-molecule BRD inhibitor (JQ1) was purchased from Selleck Chemicals and dissolved in DMSO. Zebrafish of mixed genders with the almost same tumor burden were treated with JQ1 at 4 μM (*n* = 5) or with vehicle (DMSO) (*n* = 5) in individual tanks. Zebrafish were treated with JQ1 at 4 μM in individual tanks. The treatment was conducted for the total duration of 15 days. Water containing JQ1 was changed every 48 h.

### Transplantation assay

Tumor cells (*n* = 100,000) were transplanted into the peritoneal cavity of 3-month-old *rag2*^*E450fs*^ mutant fish of mixed gender, as previously described^78^.

### Histopathology

Adult zebrafish were euthanized, fixed in 10% neutral-buffered formalin, decalcified with 9% EDTA (pH 7.6), dehydrated in a graded ethanol series, cleared with Histo-Clear (Sigma Aldrich), and embedded in paraffin. After rehydration using Histo-Clear and a graded series ethanol, the section slides were stained using hematoxylin and eosin (H&E). High-magnification imaging was performed, and images were acquired using Axioplan 2 Imaging (Zeiss) and AxioVision software. Macroimages were taken using an Olympus SZX fluorescent stereo zoom microscope, and Olympus DP software was used for image acquisition.

### Flow cytometry

Cells were prepared from wild-type adult zebrafish kidney and mCherry-sorted cells from the Tg(*lck:IRF4;p53*^*wt/wt*^) fish. Cells were gated on FSC-H and FSC-A plot to eliminate doublets and then on FSC versus SSC plot to obtain hematopoietic lineage profiles for erythrocytes, granulocytes and monocytes, lymphocytes, and blood cell precursors. FSC Forward scatter, FSC-H Forward scatter height, FSC-A Forward scatter area, SSC Side scatter.

### Genotyping

Adult fish were anesthetized in MS-222 solution and fin clipping was performed. To extract genomic DNA, the fin tissue was lysed in 50 µL of NaOH (50 mM) and incubated at 95 °C for 1 h in a thermocycler. The reaction was then neutralized with 5 µL of Tris-HCl (1 M) and vortexed briefly. 1 µL of genomic DNA or 100 ng of plasmid control were subjected to PCR amplification using Accuprime™ Pfx DNA polymerase (Thermo Fisher Scientific). The PCR cycle parameters were as follows: initial denaturation at 95 °C for 3 min, and 34 cycles of (i) denaturation at 95 °C for 30 s, (ii) annealing at 59 °C for 30 s, (iii) elongation at 72 °C for 1 min 30 s, followed by a final elongation step at 72 °C for 5 min. The primers used for genotyping are shown in Table 1.

**Table 1:** Primer sequences used for genotyping analysis.

Primer ID	Primer sequence (5′ ––>3′)
IRF4 CDS forward CACC	5′-CACCATGAACCTGGAGGGC-3′
IRF4 CDS reverse	5′-TCATTCTTGAATAGAGGAATGGCGG-3′
mCherry AgeI forward	5′-AATACCGGTATGGTGAGCAAGGG-3′
mCherry ClaI reverse	5′-CCATCGATCTACTTGTACAGCTCGT-3′
rag2 forward	5′-CTGCCGGATCTCCCATGGAC-3′
rag2 reverse	5′-TCATTCTTGAATAGAGGAATGGCGG-3′

### RNA extraction and quantitative reverse-transcription PCR (qRT-PCR)

Total RNA was isolated from the sorted mCherry cells from each transgenic zebrafish using a NucleoSpin RNA kit (Macherey-Nagel). RNA was reverse-transcribed using EvoScript Universal cDNA Master Mix (Roche). qRT-PCR was performed on a QuantStudio 5 Real-Time PCR system (Thermo Fisher Scientific) using SYBR Green PCR master mix (Applied Biosystems) and analyzed using QuantStudio^TM^ Design and Analysis software version 1.2. The primer sequences are shown in Table 2.

**Table 2:** Primers used for RT-PCR.

Genes	Species	Forward primers	Reverse primers
IRF4	Human	GGCCCAGCTTGTGAAAATGG	TTATGCTTGGCTCTGTGGGG
trac	Zebrafish	CTTAAAACGTCGGCTGTCCG	TGAACAAACGCCTGTCTCCT
trbc	Zebrafish	CTCCGGAAAAAAGTCACACTTG	AAGATGACAAGGCCATACAGTC
trgc	Zebrafish	TAGTAACTGAACCTGGGAAGGAC	TTAAGAGCGCTCCTCTTTCTTTCC
trdc	Zebrafish	GTGGCCGCCGGATTCTTTCCTCA	TTTGTGGATGGTGGGGTGGTAGT
cd4-1	Zebrafish	TCAACACCAAGACCATCAGC	GCACATGTCCATTTCACCTC
cd8a	Zebrafish	TCGGAGGTTGTGGACTTTTC	TTGTAATGGTGGGGACATCG
gata3	Zebrafish	GGTGAGATGTAGGGAGAGGAAACC	TGCCCAAGACCTATAACACATCCA
mycb	Zebrafish	AAGCGGCCAAAGTGGTGATCC	CACTACTTTGCCACACCCTCGC
trgc	Zebrafish		GAGCACAGCCACAATGAAGA
trgv1	Zebrafish	GCCCCATCAAGACTGCTTTA	
trgv2	Zebrafish	AGATGGTGCTGTTGTTCGTGA	
trgv3	Zebrafish	GCGTTCTGGGAGTGACTTTG	
trgv5	Zebrafish	CTCAGAAGGTGTTTGTGAAGC	
trgv6	Zebrafish	ACCAAATCTCAGGACAAGACTG	
trgv7	Zebrafish	ATGCACAAATTTCAGGAGGAGA	
si:dkeyp-13d12.11	Zebrafish	ATCAAATGCCAAGTGGATGC	
si:dkeyp-13d12.24	Zebrafish	ATCAAATGGACCGTGGAATC	
midn	Human	AGAGCCAGATCCGCATGTGCAA	TTCTACGGAGCCGTTTCTGCTG
patl1	Human	GCCAAACTGGAGCACGCCTATA	CACTCCGAGATGTCACAACAGC
psip1	Human	AGGCAGGAGTAGTGACAACAGC	CTCTCTGAAGGACAGGGCTGTT

### Cell line and overexpression experiments

A human cell line (HPB-ALL) was purchased from DSMZ and used from the stock confirmed by DNA fingerprinting using the PowerPlex 1.2 system (Promega) in January 2013. The cells were cultured in Roswell Park Memorial Institute (RPMI)-1640 medium (BioWest) supplemented with 10% fetal bovine serum (FBS)(BioWest). 293 T cells were cultured in Dulbecco’s modified Eagle’s medium (DMEM) supplemented with 10% FBS, penicillin (100 U/mL), and streptomycin (100 U/mL). All cells were cultured in a humidified incubator containing 5% CO_2_ at 37 °C. For gene overexpression, the full-length human *IRF4* sequence were cloned into a doxycycline-inducible pCW57.1 lentiviral vector (Addgene). Cells were transduced with lentivirus produced by cotransfection into 293 T cells with pMDLg/pRRE, pRSV-Rev, and pMD2.G using FuGENE6 (Promega) and Opti-MEM (Thermo Fisher) reagents.

### Protein extraction and Western blotting

FACS-sorted cells were homogenized in lysis buffer containing 20 mM Tris-HCl (pH 7.5), 150 mM NaCl, 1 mM Na_2_EDTA, 1 mM EGTA, 1% Triton, 2.5 mM sodium pyrophosphate, 1 mM β-glycerophosphate, 1 mM Na_3_VO_4_, and 1 μg/ml leupeptin (Cell Signaling Technology) supplemented with 1% Halt protease inhibitor cocktail (Thermo Fisher Scientific). Protein concentrations were then quantified using a Bio-Rad protein assay kit (Bio-Rad Laboratories). A total of 15 µg of each protein lysate from mCherry-positive cells of the Tg*(lck:IRF4)* fish were mixed with 4× Laemmli sample buffer (Bio-Rad Laboratories) and β-mercaptoethanol (Sigma-Aldrich) in appropriate proportions before being incubated at 99 °C for 5 min. The samples were then separated on SDS-PAGE gels and transferred to PVDF membranes (Millipore) at 400 mA for 1.5 h, blocked with 3% skim milk (Nacalai Tesque) in ﻿Tris-buffered saline with 0.1% Tween and probed with IRF4 antibody (1:1000; Santa Cruz), cleaved PARP antibody (1: 1000; Cell Signaling Technology) and GAPDH-HRP antibody (1: 1000; Santa Cruz). Secondary detection was performed with HRP-linked antirabbit IgG (Cell Signaling Technology) and ECL™ Western blotting detection reagents (Fisher Scientific). Western blot images were captured using an ImageQuant LAS500 chemiluminescent image analyzer (GE Healthcare).

### Single-cell RNA sequencing (scRNA-seq)

#### Labeling and library preparation

Samples were prepared according to the 10x Genomics Single Cell 3′ v2 Reagent Kit user guide as previously described^79^. Briefly, mCherry-positive cells were first sorted by flow cytometry from the whole body of leukemic zebrafish. Cell viability was evaluated using trypan blue and a hemocytometer. Samples were prepared for target capture of 6,000 cells. Gel beads in emulsions (GEMs) were prepared by combining gel beads containing barcoded oligonucleotides with partitioning oil and were loaded onto the microfluidic chip. Then, the gel beads were dissolved, and cDNA libraries were constructed as described by the Single Cell 3′ Reagent Kit v2 user’s guide with appropriate modifications to the PCR cycle based on the calculated cDNA concentration.

#### Sequencing

The final library was amplified and sequenced using the P5 and P7 primers in an Illumina HiSeq at Novogene AIT Genomics Pte Ltd. The library was sequenced in a paired-end and non-strand-specific manner with a 150-bp read length.

#### Single-cell data analysis

Single-cell sequencing data were processed using the 10x Genomics software package CellRanger v3.1.0. Flow of data analysis is shown in Supplementary Fig. 7. The reads were aligned to the GRCz11/danRer11 genome, and *mCherry*, human *IRF4* (ENSG00000137265), the TCR-α constant (*trac)* and β constant (*trbc*) genes reported in Wan et al.^42^ were manually included in the annotation. Constructs generated using the consensus *v-j-c* sequence from zebrafish *trgv1*-*trgv7* reported by Beetz and Steiner in Nucleotide Database (NUCCORE: https://www.ncbi.nlm.nih.gov/nuccore) were also manually included. Zebrafish transcriptome data were generated by filtering the Ensembl annotations for protein-coding immunoglobulin and TCR genes that were defined in the GRCz11 GTF file. Individual read counts were performed using the CellRanger “count” function, which counted the reads based on the number of observed cell barcodes and generated a matrix for each cell and count. Then, all the samples (*n* = 20) were combined into a single aggregated version to determine gene expression using both the CellRanger “aggr” function and Seurat R package v3.0^80^ and were visualized using Loupe Browser v5.0 software. QC metrics showing the number of cells after each filtering were shown in Supplementary Data 5.

#### Data integration

Doublet removal was performed by integrating the outputs of DoubletFinder^81^ and DoubletDecon^82^. The R package Seurat v3.0 was used to filter, integrate and merge the data^83^. Each sample was filtered with gene features >500 and mCherrry expression ≥1 UMI. The anchors of each sample were determined by assigning each anchor a score and constructing an overall neighbor graph and computing their SNN graph using the FindIntegrationAnchors function (*n* = 3,000). Later, the distance between datasets were defined by the number of anchors between datasets, the pairwise distance between datasets were computed and clustered to determine a guide tree. These integrated multiple datasets (*n* = 20) into a single merge Seurat object by using the IntegrateData function. Principal component analysis (PCA) was performed using the RunPCA function with 20 dimensions (dims = 20), followed by uniform manifold approximation and projection (UMAP) visualization of the PCA data using the RunUMAP function. The annotation was firstly done using the control fish scRNA-seq by exploring the marker genes for each population. Cell populations were defined based on the marker gene expression (Supplementary Data 1). Because the expression pattern of hematopoietic cell markers can be different among species, we used only the genes that have been reported to be expressed in zebrafish^37–41^. Then, the same marker genes were applied to the rest of the individual tumor sample to classify their cell population. The single-cell plot were visualized using the DimPlot function on the UMAP reduction, and the gene expression plot was visualized using the FeaturePlot function. All these functions are available in the Seurat R package v3.0. Violin plots, and gene expression images were generated using Loupe Browser v5.0. All TCR-γ variable genes (trgv1-trgv7) were calculated using AddModuleScore by summing the manually constructed *v-j-c* counts and visualized using FeaturePlot. All these functions are available in Seurat R package v3.0.

#### Single-cell differential analysis

Significant marker genes were selected by comparing one cluster to all the other clusters using Loupe Browser v5.0 with the ‘locally distinguishing’ method to measure the significant genes in each cluster. Loupe Browser uses the sSeq methods for differential expression analysis which employs the negative binomial exact test. Differential expression data were retrieved from the average expression of each gene per sample and the heatmap plot using ComplexHeatmap R package version 2.1.0^84^. The *p*-values were adjusted for multiple testing using the Benjamini-Hochberg procedure and were generated by the Loupe Browser.

### Chromatin-immunoprecipitation (ChIP)-sequencing (ChIP-seq)

Tumor cells and normal thymus samples at 3 months were sorted for mCherry-positive cells, crosslinked and processed for ChIP as described previously^85^. Sonicated chromatin was incubated with Dynabeads (Invitrogen) coated with H3K27ac (Abcam, #ab4729). Library construction and BGISEQ-500 sequencing of single-end, 50-bp reads were performed at BGI Biotech Solutions Co. Ltd. (Hong Kong). ChIP-seq reads were mapped to zebrafish (Danio rerio GRCz11) reference genome using Bowtie2 version v2.4.1 program^86^ with default parameters. Aligned ChIP-seq reads were processed with samtools v1.12 rmdup package to remove duplicates. ChIP-Seq peaks were called with MACS2 software, version 2.1.1.2016030. Threshold for the broad peaks called is *P*-value < 1E-3 and the broad peaks cutoff is *P*-value < 1e-3 –broad-cutoff 1e-3 -p 1e-3 –broad –keep-dup 1 -f BAM –SPMR. Differential binding analysis was performed using DiffBind v3.0. The peaks of each sample were merged and consensus sites between all samples (4 tumor vs. 3 normal thymus) were compared using DESeq2 algorithm^87^ in DiffBind R package^88^. The significant regions (FDR <0.05) reported by DiffBind were selected and grouped into Group I (log2FC >1), Group II (1 < log2FC > −1) and Group III (log2FC < −1). 500 bp from the center of each significant region (250 bp to both ends) were extracted to perform motif enrichment analysis. Super-enhancers were identified by ROSE2 version 1.1.0 by merging peaks less than 12.5 kb and excluding signal from 2 kb of gene promoter reported in GRCz11 Ensembl annotation. Super-enhancers were called with the parameter -s 12500 -t 2000 –mask hg19_blacklist.bed. Closest genes were annotated for each super-enhancer and top gene were reported by ROSE2 annotation by analyzing each closest gene with highest promoter H3K27ac signal and selected to be the top gene. Motif enrichment analysis using meme-chip function from MEME Suite^89^ by utilizing the vertebrates PWN from Transfac and HOCOMOCO v11 motif database.

### Bulk RNA-seq

Tumor fish were treated with JQ1 (4uM) or control DMSO for 3 days. Tumor cells were sorted by flow cytometry based on mCherry fluorescence expression. Total RNA was harvested using the miRNeasy Mini Kit (Qiagen). ERCC spike-in was added based on the total cell number. Strand-specific library construction and sequencing of paired-end, 100-bp-long reads by the BGISeq500 were performed at the BGI Biotech Solutions (Hong Kong) Co Ltd (Hong Kong). chRNA-seq datasets of DMSO-treated control samples and JQ1-treated samples were aligned to the GRCz11 danRer11 genome Ensembl annotation with ERCC spike-in information using STAR 2.5.2a^90^ with the parameter outFilterMultimapNmax set to 1. FeatureCount was used for the mapped reads in bam files to generate count tables based on the Ensembl gene annotation and ERCC annotation. Bioconductor R package DESeq2 v1.12.4^87^ was used to analyze differential gene expression using 3 DMSO vs 3 JQ1-treated samples. RNA abundance quantification in GD-TCL patients and normal GD-T cells were quantified using the kallisto software version 0.45^91^ with the following parameter -b 30.

### Gene expression in mouse hematopoietic cells

The mRNA expression pattern in various stages and lineages of mouse hematopoietic cells were analyzed using Gene Expression Commons database^92^.

### Statistical analysis and reproducibility

The significance (*p* values) of the tumor monitoring analysis was determined using the Gehan-Breslow-Wilcoxon test in GraphPad Prism software. For the significance of gene expression analysis of scRNA-seq data, *p* values adjusted for multiple testing using the Benjamini-Hochberg procedure were generated by the Loupe Browser. The significance of other analyses was determined using the Student’s *t*-test in GraphPad Prism software. *F*-test was performed to analyze the variances of two populations, based on which one or two-tailed test was selected. A *p* value less than 0.05 was considered statistically significant. For microscopic and histopathological analyses, we showed representative images from multiple independent animals. Animal numbers are indicated in the legend.

### Reporting summary

Further information on research design is available in the Nature Research Reporting Summary linked to this article.

## Supplementary information

Supplementary Information

Description of Additional Supplementary Files

Supplementary Data 1

Supplementary Data 2

Supplementary Data 3

Supplementary Data 4

Supplementary Data 5

Reporting Summary

## Data Availability

The regular RNA-seq, ChIP-seq and scRNA-seq data generated in this study have been deposited in the Gene Expression Omnibus database: accession numbers GSE139226 (RNA-seq, publicly available), GSE166644 (ChIP-seq, publicly available), GSE166646 (scRNA-seq, publicly available), GSE184946 (RNA-seq, publicly available) under the super-series GSE166650. The dataset for human γδ T-cell lymphoma and normal γδ T-cells has been reported^52^ and deposited under the dbGaP and GEO databases under accession number phs001969 (RNA-seq, publicly available) and GSE107011 (RNA-seq, publicly available). The dataset for *rag2-Myc* zebrafish has been reported^47^ and deposited under GEO database under accession number GSE108855 (RNA-seq, publicly available). Zebrafish genome and annotation version GRCz11 [http://asia.ensembl.org/Danio_rerio/Info/Index] is available from Ensembl database. Zebrafish raw images have been deposited in figshare (10.6084/m9.figshare.19497848, publicly available) [10.6084/m9.figshare.19497848]. Source data are provided with this paper.

## Source data

Source Data
