# Supplementary material for: IRF4 drives clonal evolution and lineage choice in a zebrafish model of T-cell lymphoma
Source: Nat Commun. 2022 May 3;13:2420. doi: 10.1038/s41467-022-30053-9 (PMC9065160; doi:10.1038/s41467-022-30053-9)
Supplement: Supplementary file 1 — Supplementary Information [file 41467_2022_30053_MOESM1_ESM.pdf]

A

IRF4 sequence conservation

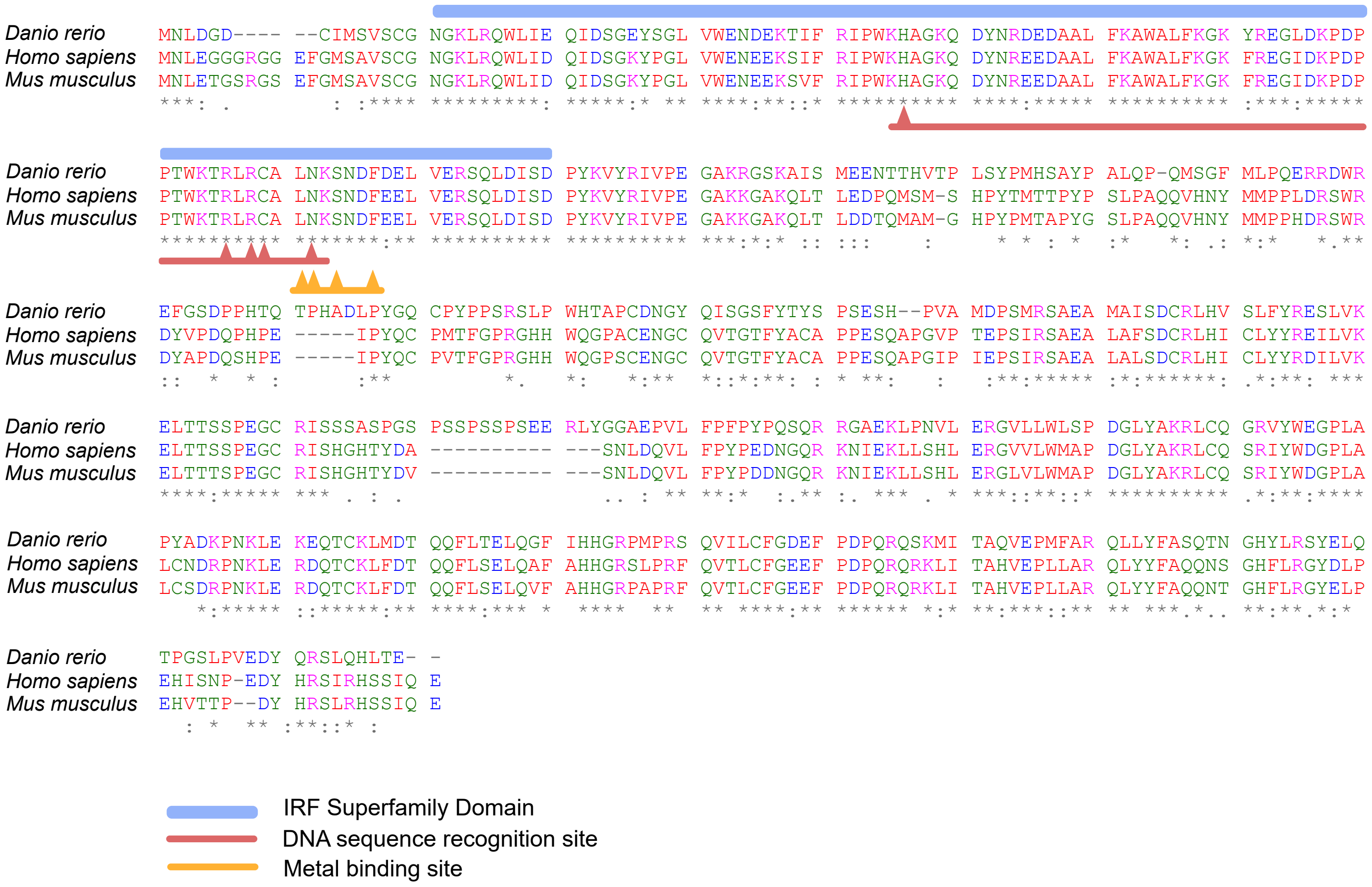

B

Mouse *Lck* mRNA

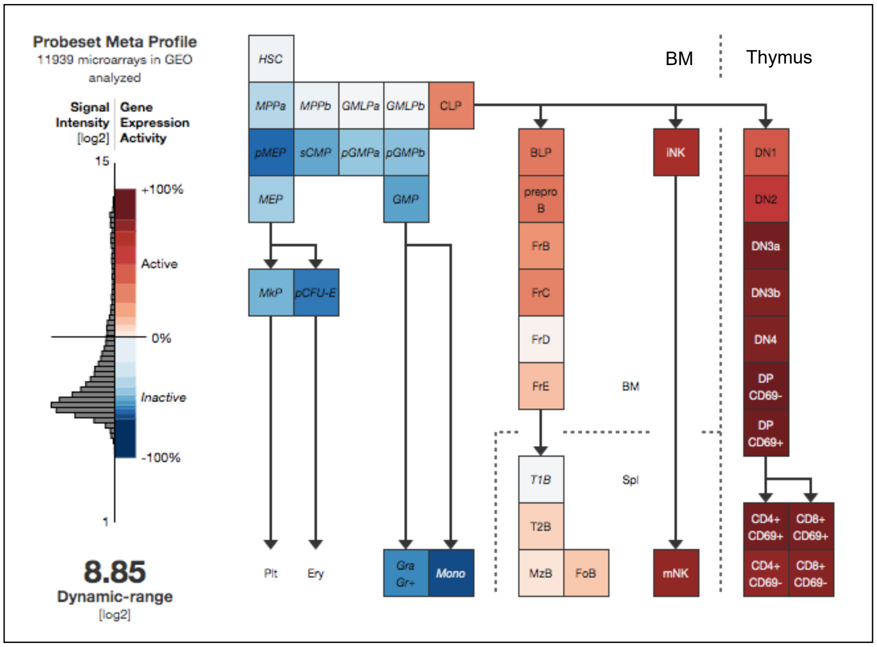

Mouse *Rag2* mRNA

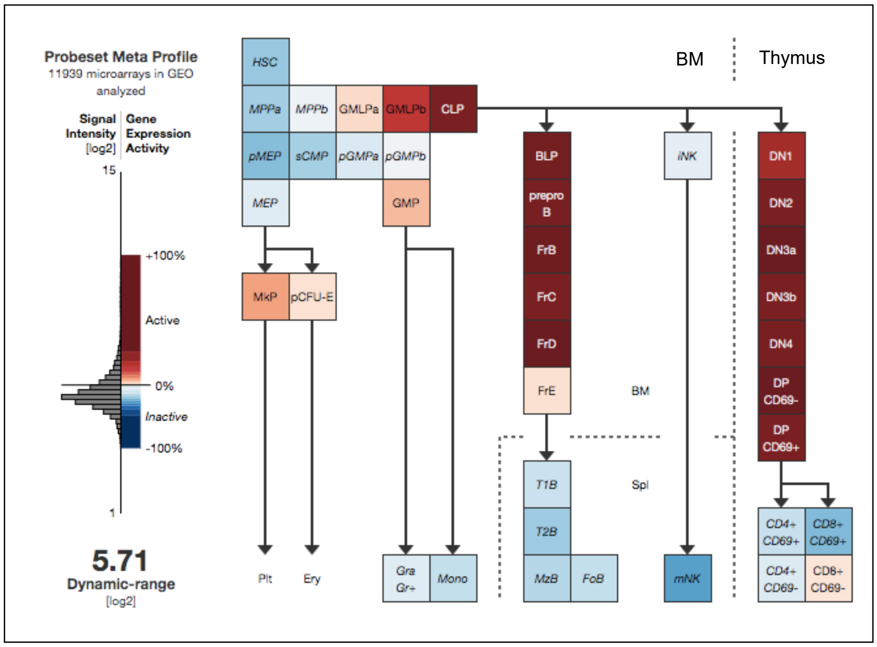

C

Zebrafish *lck* mRNA

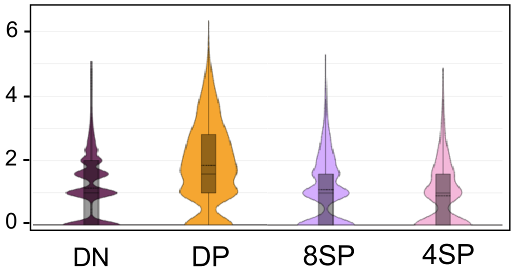

Zebrafish *rag2* mRNA

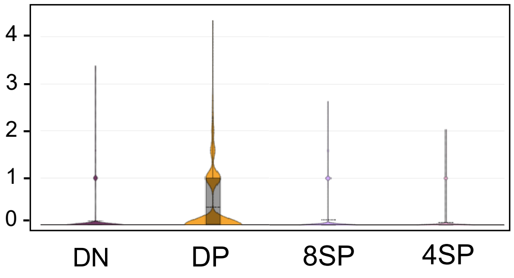

Supplementary Figure 1

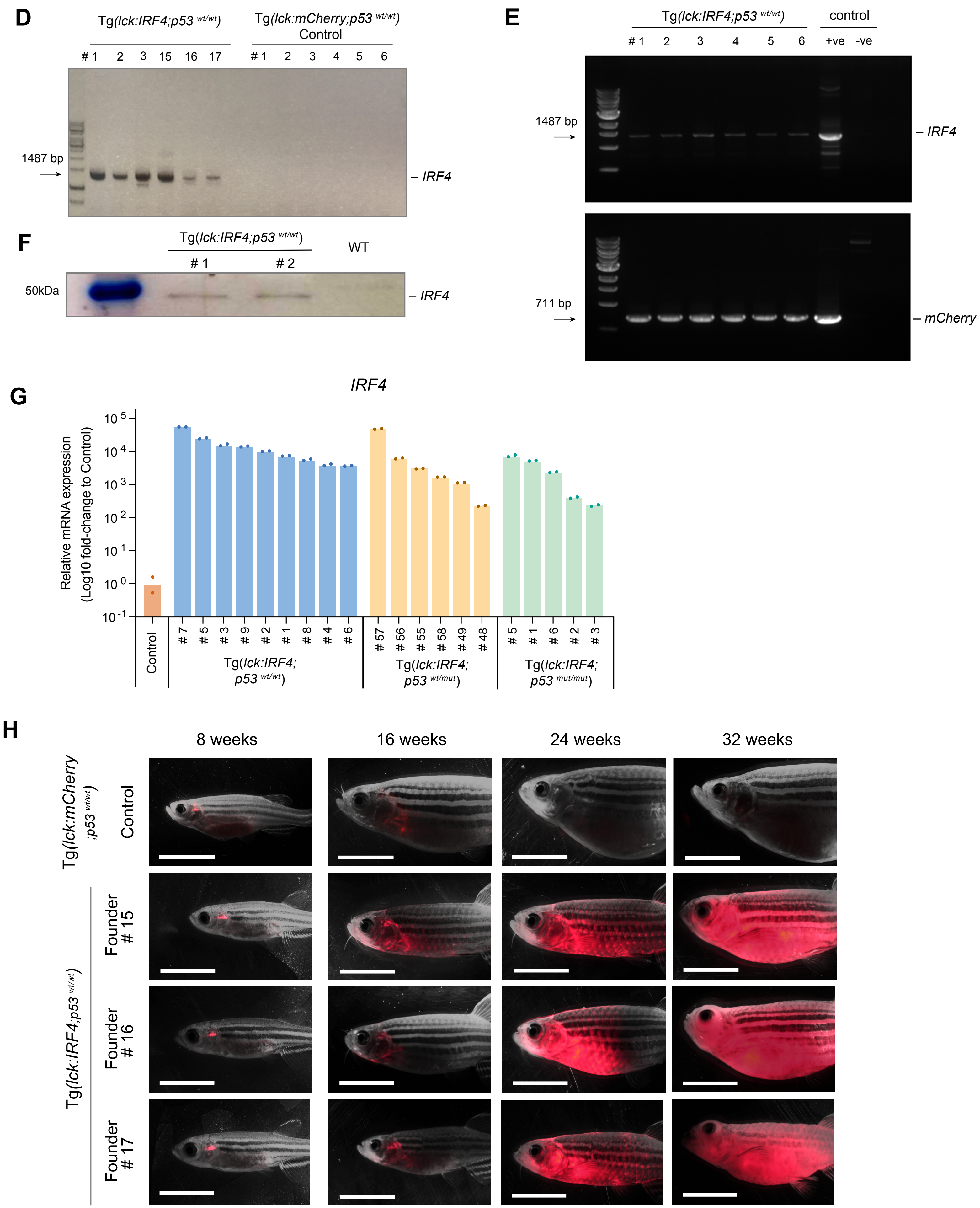

Supplementary Figure 1

I

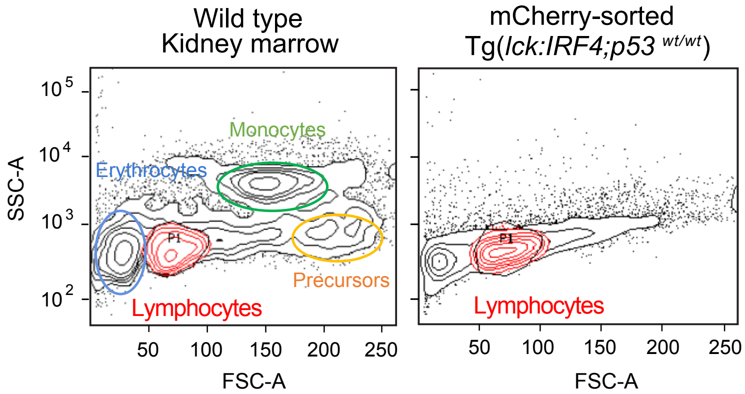

J

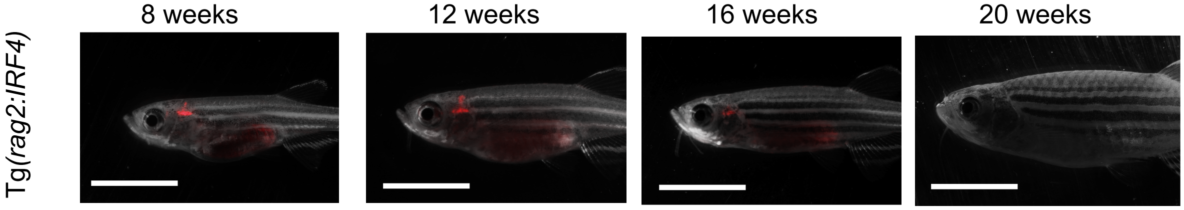

K

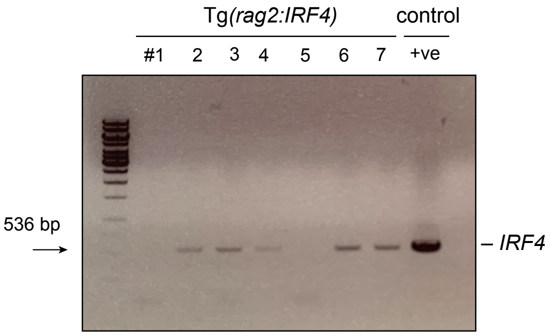

Supplementary Figure 1

Supplementary Fig. 1. Overexpression of wild-type *IRF4* induces tumors in zebrafish. (A) DNA sequence comparison showing the zebrafish *irf4* gene shares >60% amino acid identity with the human and mouse genes, while the DNA recognition site is completely conserved between species. **(B)** Heatmap depicting mRNA expression of mouse *Lck* and *Rag2* genes obtained from the Gene Expression Commons database[1]*. Rag2* is primarily expressed in immature stages of T- and B-cells, while *Lck* is expressed in both immature and mature stages of T- and B-cells. **(C)** Violin plots showing mRNA expression of zebrafish *lck* and *rag2* in different states of lymphocytes in a control fish analyzed by scRNA-seq (DN n=557 cells; DP n=1,042 cells; 8SP n=601; 4SP n=393 cells). See Fig.5C legend for the details of violin plots. **(D,E)** Genomic DNA extracted from the F0 Tg(*lck:IRF4;p53wt/wt*) and control Tg(*lck:mCherry;p53wt/wt*) zebrafish was subjected to PCR using specific primers. Original vector plasmid (ve) was used as a positive control. **(F)** Protein expression of human IRF4 from two Tg(*lck:IRF4;p53wt/wt*) zebrafish (mCherry-positive cells) and wild-type animals (whole body). Whole-cell lysates were subjected to immunoblot analysis with anti-human IRF4 antibody. **(G)** The mRNA expression of human *IRF4* in thymic samples from control Tg(*lck:mCherry;p53wt/wt*)*,* nine Tg(*lck:IRF4;p53wt/wt*)*,* six Tg(*lck:IRF4;p53wt/mut*) and five Tg(*lck:IRF4;p53mut/mut*) zebrafish as determined by qRT-PCR in technical duplicates. The gene expression levels were normalized to that of the ERCC spike-in (internal control). Because these are technical duplicate samples, statistical analysis is not performed. **(H)** Representative microscopy images of the control Tg(*lck:mCherry;p53wt/wt*) and Tg(*lck:IRF4;p53wt/wt*) F0 founder zebrafish. Panels show merged fluorescence and brightfield images. Scale bar = 4 mm. **(I)** Flow cytometry analysis on wild-type adult zebrafish kidney and mCherry-sorted cells from the Tg(*lck:IRF4;p53wt/wt*) fish. Cells were gated on FSC vs SSC plot to remove doublets. The gated populations are outlined. FSC-A: Forward

scatter area. SSC-A: Side scatter area. **(J)** Representative microscopy images of Tg(*rag2:IRF4;p53wt/wt*) fish. **(K)** Genotyping result of Tg(*rag2:IRF4;p53wt/wt*) fish. Source data are provided as a Source Data file.

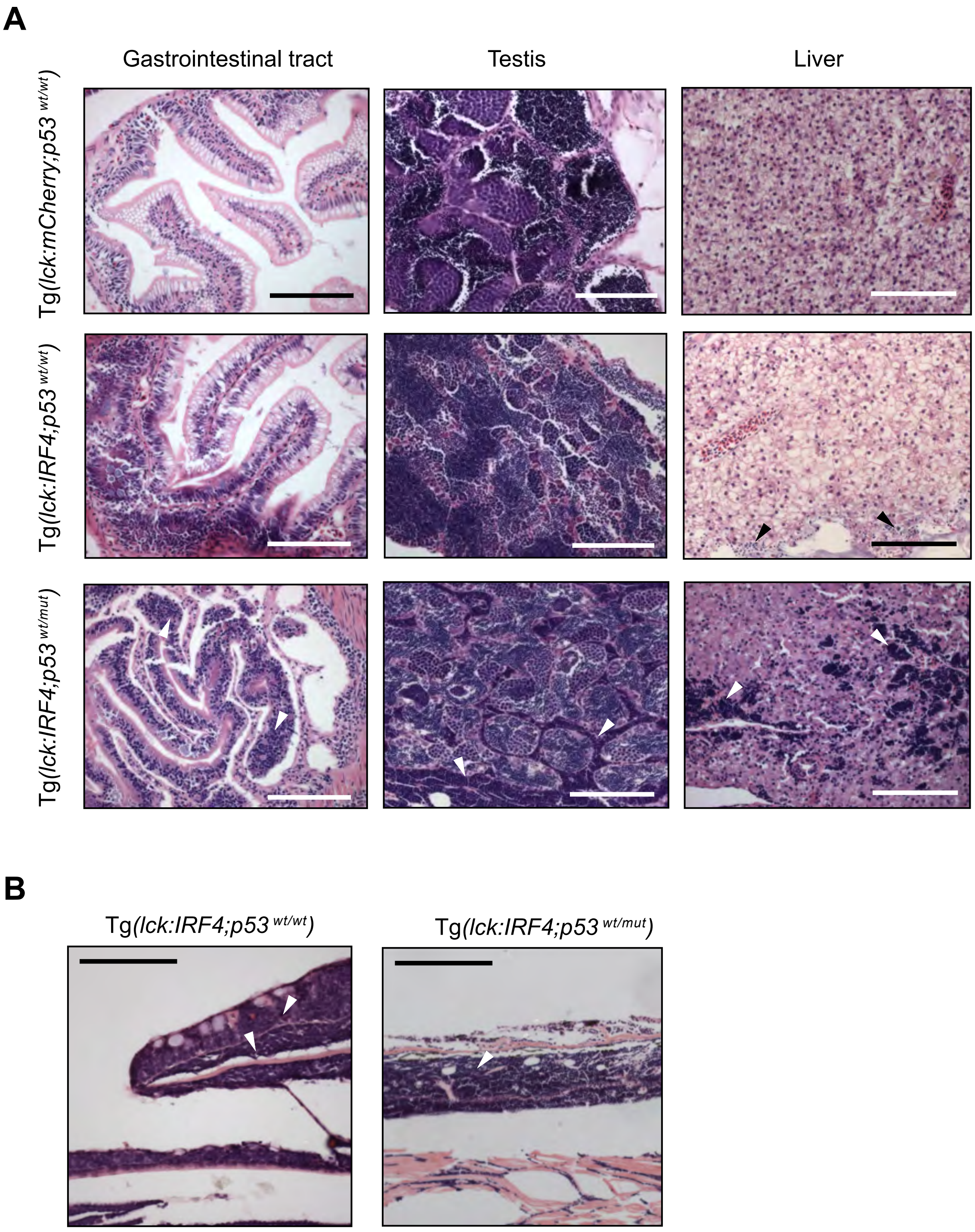

Supplementary Figure 2

Supplementary Fig. 2. *IRF4*-driven zebrafish tumors recapitulate invasive human T-cell lymphoma. (A-B) Histopathological examination of H&E-stained sections of representative samples from gastrointestinal tract, testis and liver of control Tg(*lck:mCherry;p53wt/wt*) (n=8), Tg(*lck:IRF4; p53wt/wt*) (n=8), and Tg(*lck:IRF4;p53wt/mut*) (n=4) fish (A) as well as skin of Tg(*lck:IRF4;p53wt/wt*) and Tg(*lck:IRF4;p5wt/mut*) fish (B). Tumor cells are indicated by white or black arrowheads. Similar findings were observed in multiple independent animals as shown for each sample. Imaging was performed using Axioplan 2 (Zeiss) microscope, and image acquisition was performed using AxioVision software. Scale bar = 100 μm.

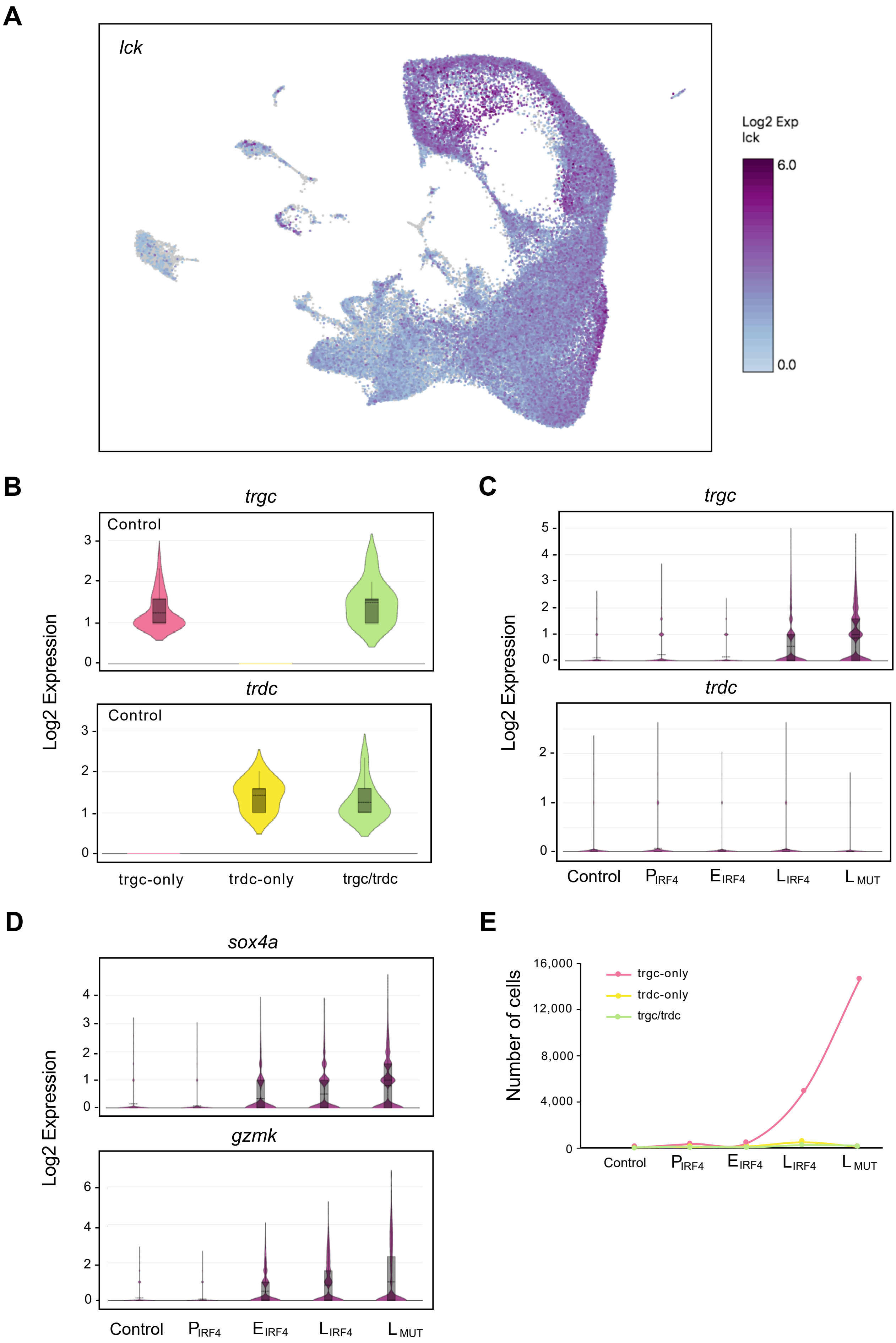

Supplementary Figure 3

F

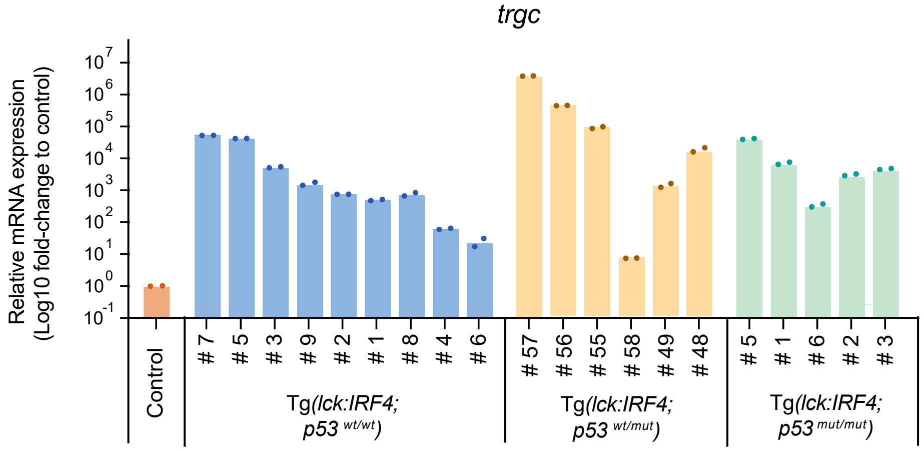

G

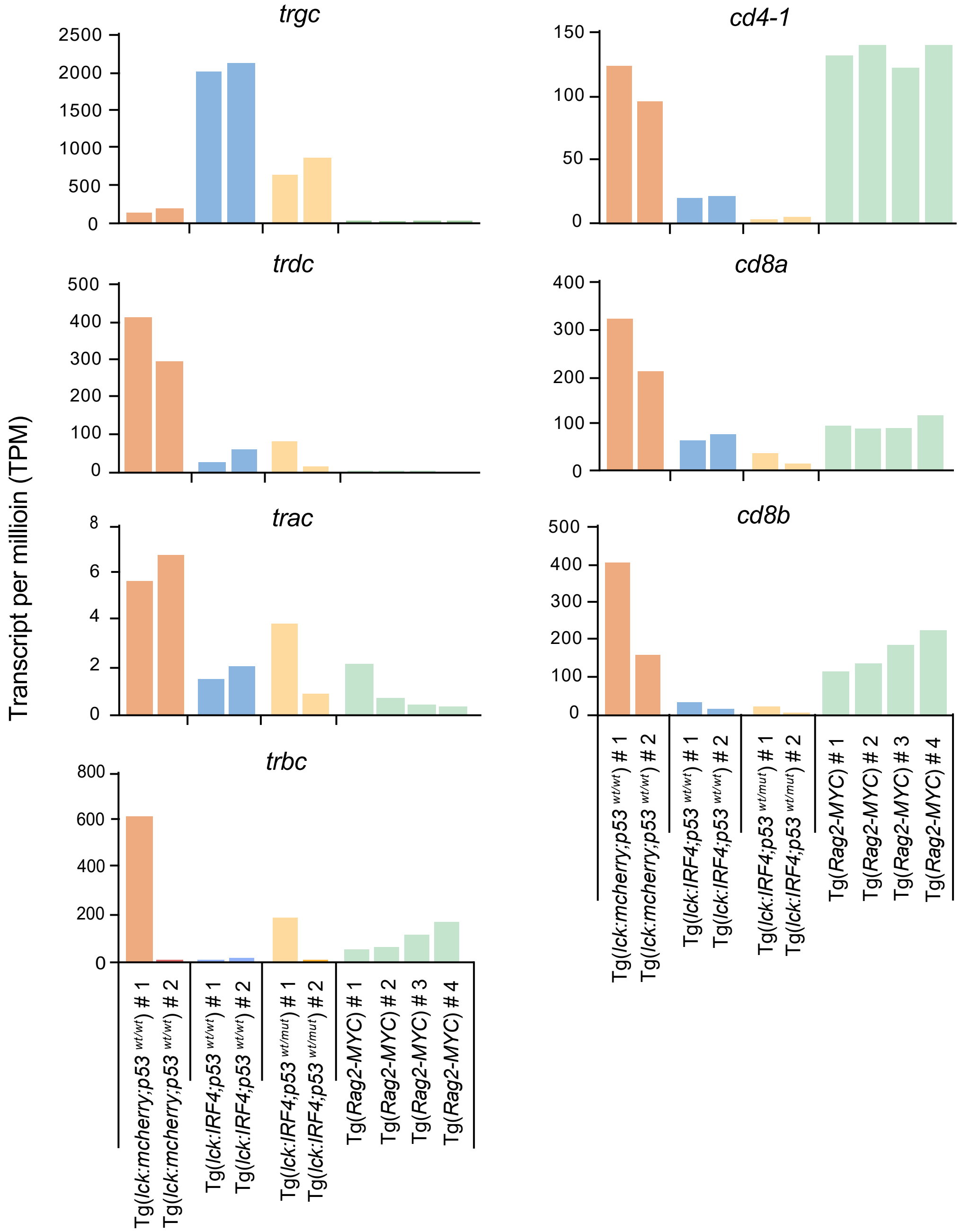

Supplementary Figure 3

Supplementary Fig. 3. Identification of multiple cell populations derived from different stages of lymphocyte differentiation. **(A)** UMAP plot showing zebrafish *lck* expression in aggregated cells from 20 independent zebrafish. Color scale indicates the expression level in log 2 scale. **(B)** Violin plots showing the expression of *trgc* and *trdc* in control analyzed by scRNA-seq in a control sample (*trgc*-only n=47 cells; *trdc*-only n=8cells; *trgc/trdc* n=10 cells). See Fig.5C legend for the details of violin plots. **(C-D)** Violin plots showing the expression of *trgc* and *trdc* (C) and *sox4a* and *gzmk* (D) in DN cell populations across tumor stages analyzed by scRNA-seq (total cell number after merging of biologically independent samples: Control n=557 cells; P_{IRF4} n=2,222 cells; E_{IRF4} n=3,365; L_{IRF4} n=15,280 cells; L_{MT} n=28,205 cells). See Fig.5C legend for the details of violin plots. **(E)** Graph showing the number of cells in three $\gamma\delta$ T-cells populations expressing *trgc* only, *trdc* only and both *trgc* and *trdc* across different tumor stages. **(F)** mRNA expression of *trgc* in thymic samples from the control Tg(*lck:mCherry;p53wt/wt*) and tumor samples from nine Tg(*lck:IRF4;p53wt/wt*), six Tg(*lck:IRF4;p53wt/mut*) and five Tg(*lck:IRF4;p53mut/mut*) zebrafish analyzed by qRT-PCR in technical duplicates. The relative gene expression levels were normalized to those of the ERCC spike-In (internal control). Because these are technical duplicate samples, statistical analysis is not performed. **(G)** mRNA expression of zebrafish *cd4-1, cd8a, cd8b,* and *tcr genes* in Tg(*lck:mCherry;p53wt/wt*) control, Tg(*lck:IRF4;p53wt/wt*), Tg(*lck:IRF4;p53wt/mut*) and *rag2-Myc* fish analyzed by RNA-seq using our dataset and GSE108855. Source data are provided as a Source Data file.

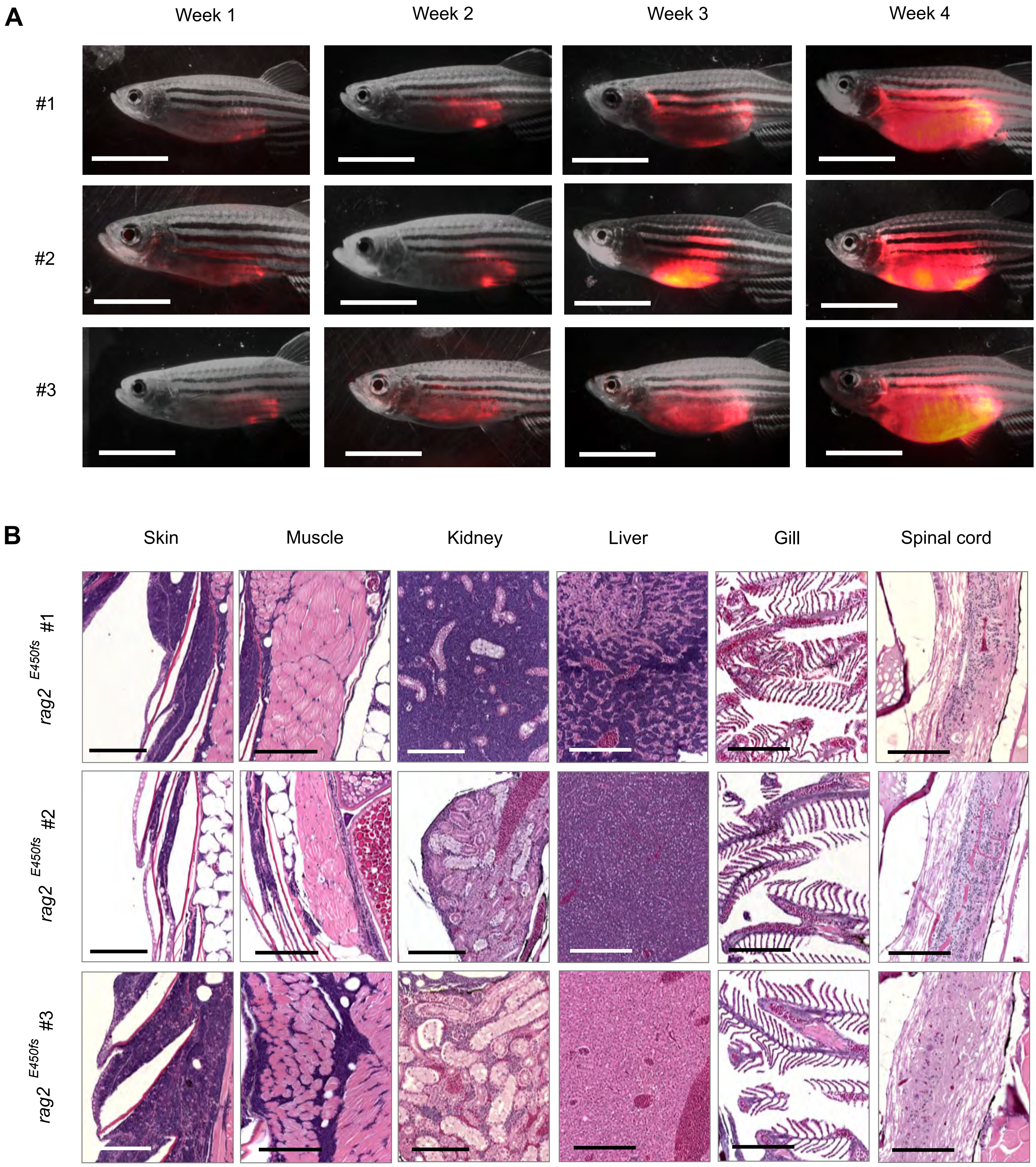

Supplementary Figure 4

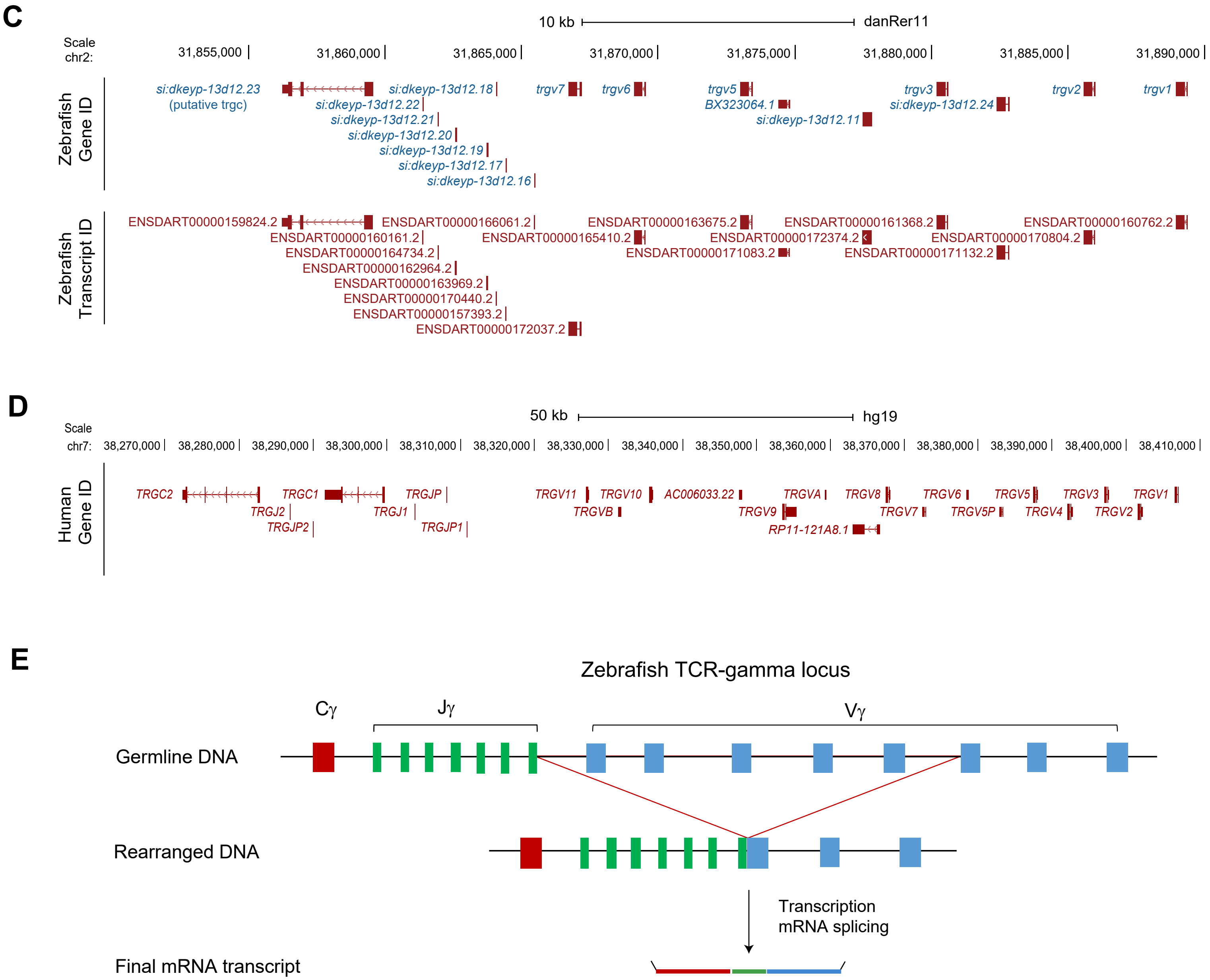

Supplementary Figure 4

Supplementary Fig. 4. IRF4-driven tumor cells possess malignant features. (A) Representative microscopy images of three recipient fish at 1, 2, 3 and 4 weeks after the second transplantation. Similar findings were observed in multiple recipient animals (n=23). See the legend of Figure 1D for details. **(B)** Histopathological examination of H&E-stained sections of representative samples from skin, muscle, kidney, liver, gill and spinal cord or three transplanted fish. Scale bar = 2 mm. Similar findings were observed in multiple independent animals as shown for each sample. **(C-D)** The gene track with Ensembl annotation showing genomic loci of the *TCR*-γ constant and variable gene regions in *Danio rerio* (C) and *Homo sapiens* (D). **(E)** Rearrangement of zebrafish *tcr*-γ locus.

A

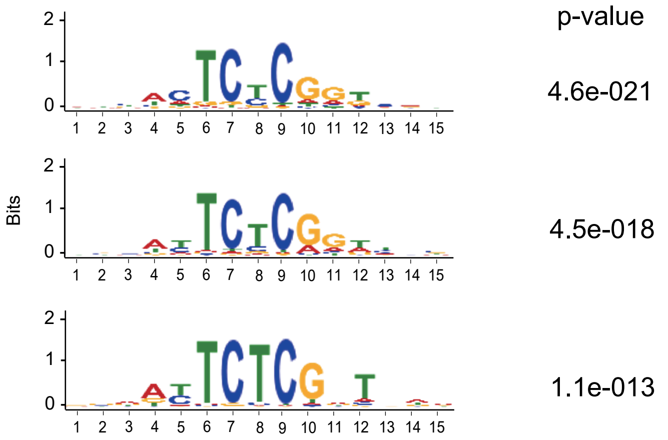

B

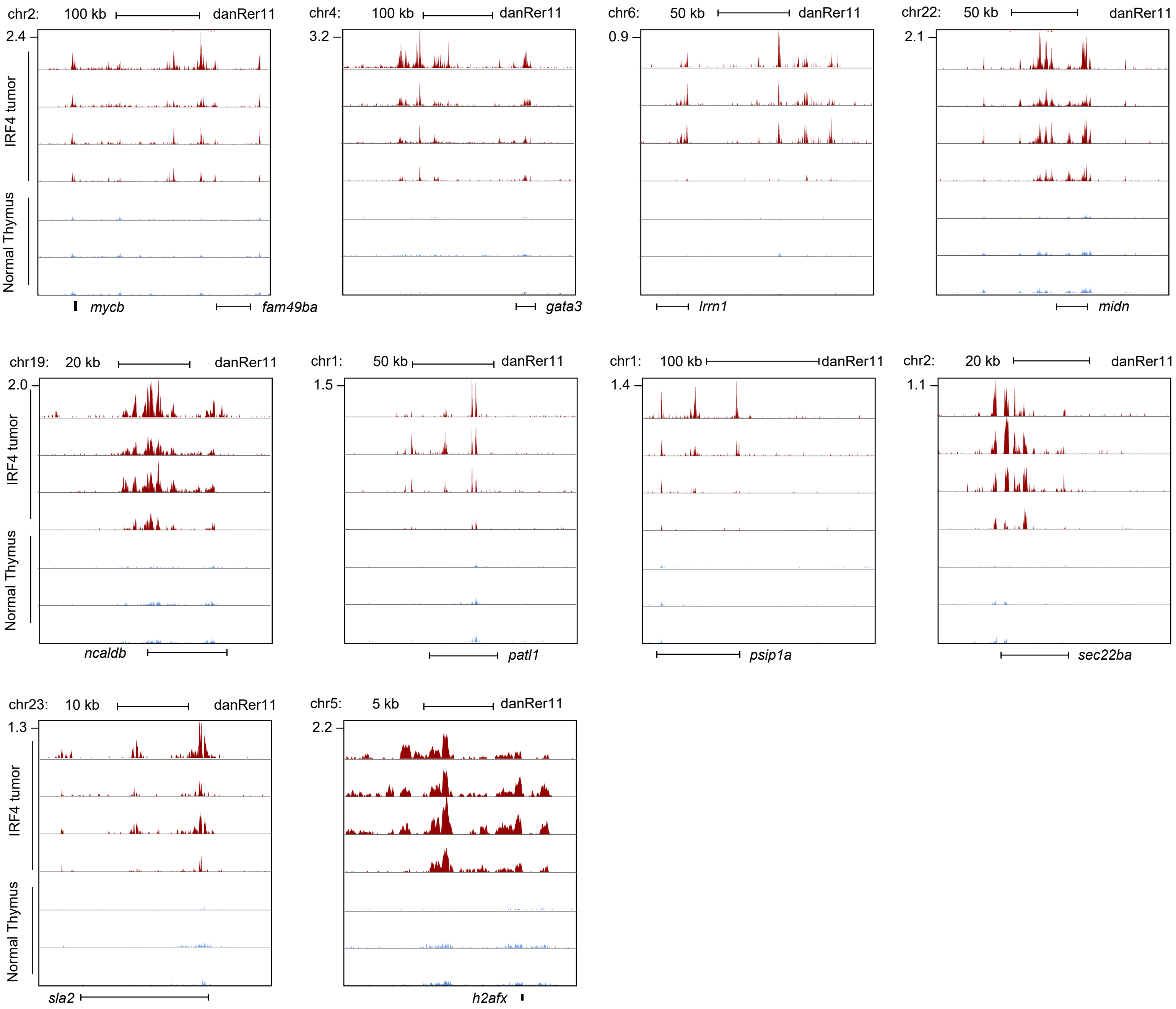

Supplementary Figure 5

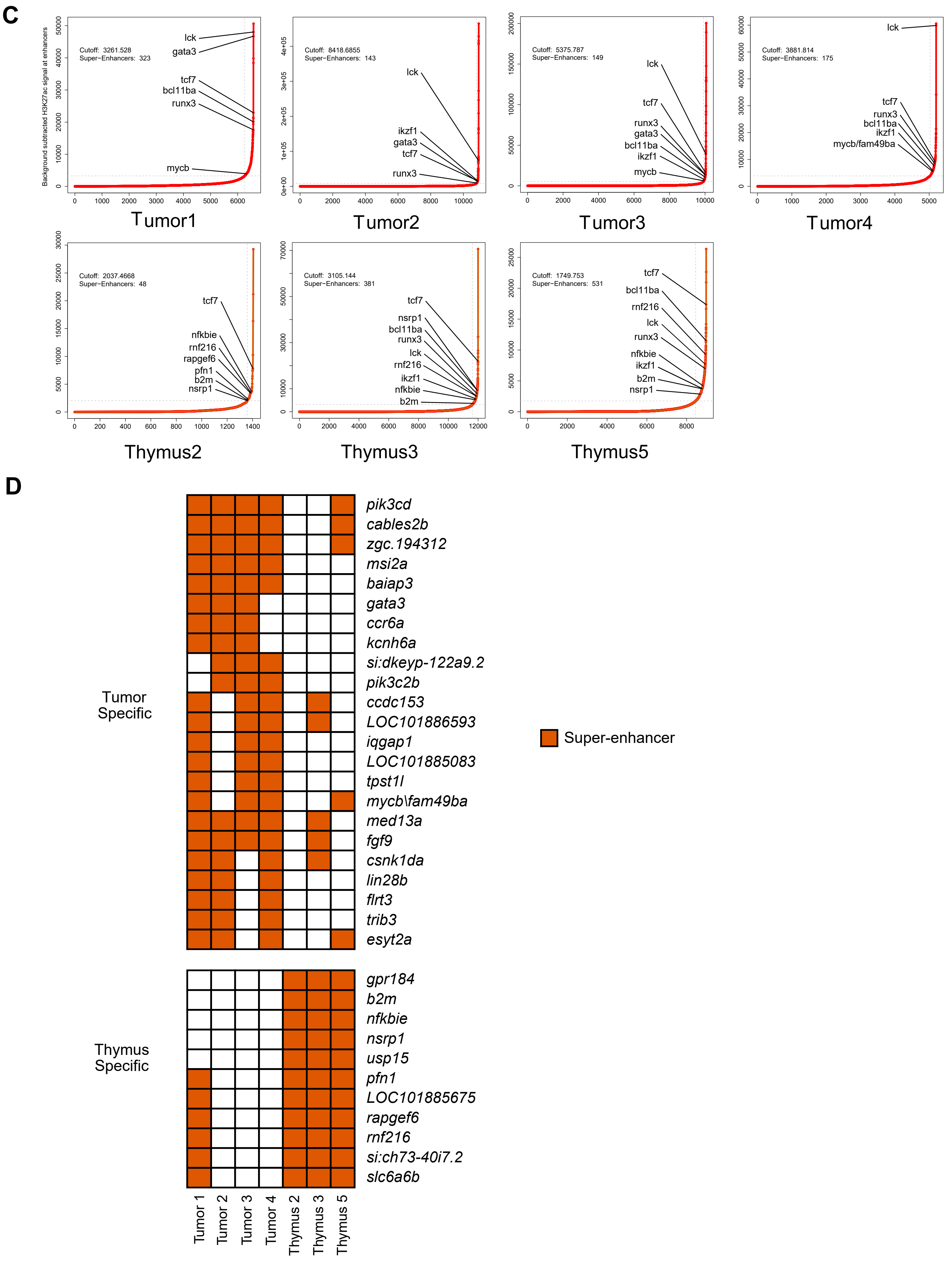

Supplemental Figure 5

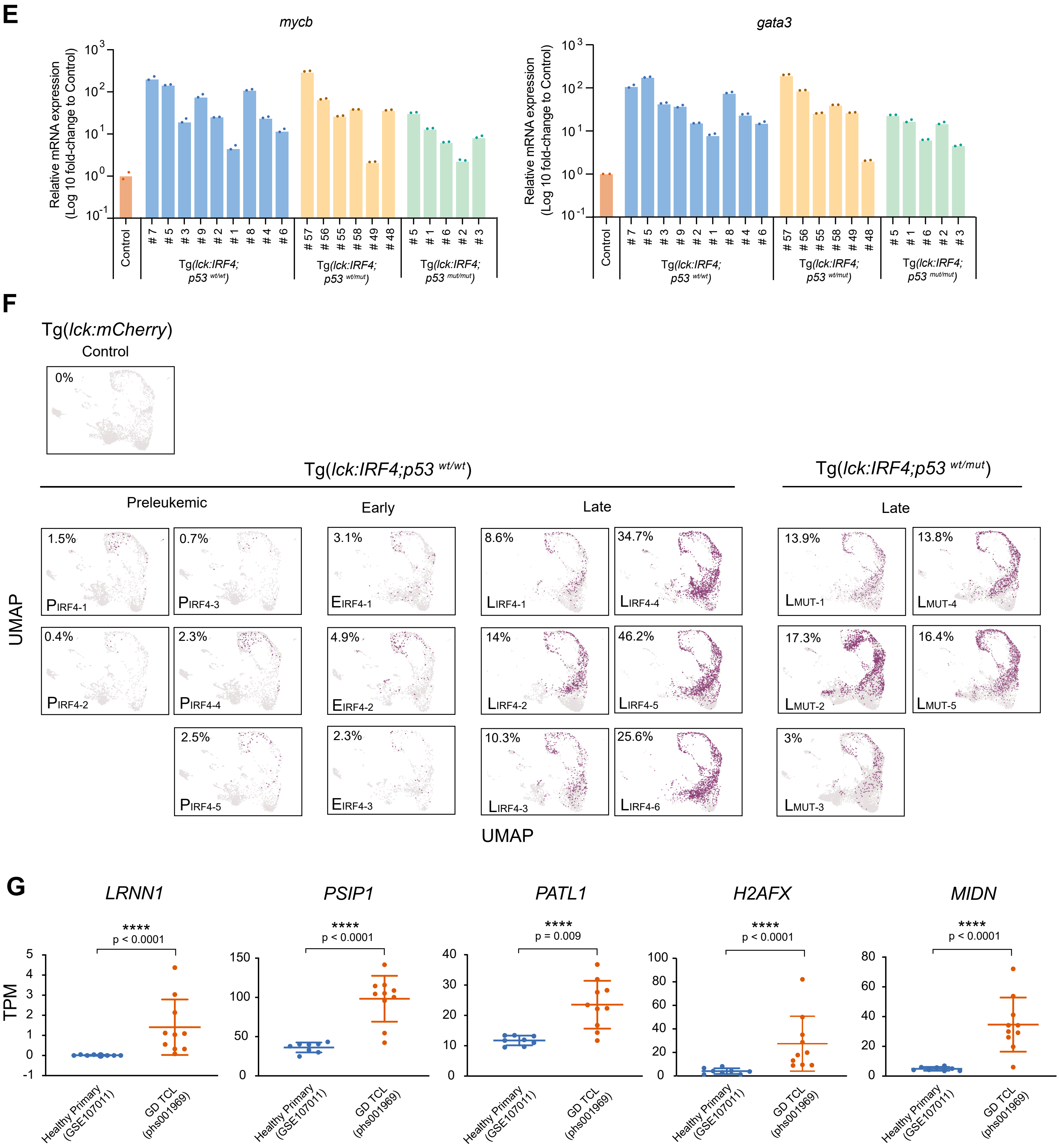

Supplemental Figure 5

Supplementary Fig. 5. IRF4-driven tumors are characterized by high expression of *mycb* and *gata3*. (A) IRF binding motifs enriched in differentially-regulated regions. **(B)** ChIP-seq gene tracks representing H3K27ac signals at ten gene loci in four zebrafish tumor samples and three pooled normal thymus samples. The x-axis indicates the linear sequence of genomic DNA, and the y-axis indicates the total number of mapped reads per million. The black boxes in the gene map represent exons, and the arrows indicate the location and direction of the gene. **(C)** Super-enhancer plots showing all super-enhancers detected by the ROSE for each sample. Representative genes which were recurrently associated with super-enhances in tumor (≥3 out of 4) or/and normal thymus (≥2 out of 3) are shown. **(D)** A table showing super-enhancers that are more frequently found in tumors (≥3 out of 4 tumors and ≤1 out of 3 thymus) and the ones that are more frequently found in thymus (3 out of 3 thymus and ≤1 out of 4 tumors). Samples with super-enhancers are shown in red. **(E)** The mRNA expression of *mycb* and *gata3* in thymic samples from control Tg(*lck:mCherry;p53wt/wt*)*,* nine Tg(*lck:IRF4;p53wt/wt*), six Tg(*lck:IRF4;p53wt/mut*) and five Tg(*lck:IRF4;p53mut/mut*) zebrafish analyzed by qRT-PCR in technical duplicates. The relative gene expression levels were normalized to those of the ERCC spike-in (internal control). Because these are technical duplicate samples, statistical analysis is not performed. **(F)** UMAP plots showing the percentage of cells that express all of *lrrn1, psip1* and *patl1* in 20 samples. **(G)** mRNA expression of five selected genes in human γδ T-cell lymphoma samples (GDTCL: n=10) and healthy primary γδ T-cells (n=8) using phs001969[3]. ***p<0.001, ****p<0.0001 by two-sample, two-tailed Student's t-tests, compared to the expression in health primary γδ T-cells. The data represent the mean ± SD of multiple independent samples. Source data are provided as a Source Data file.

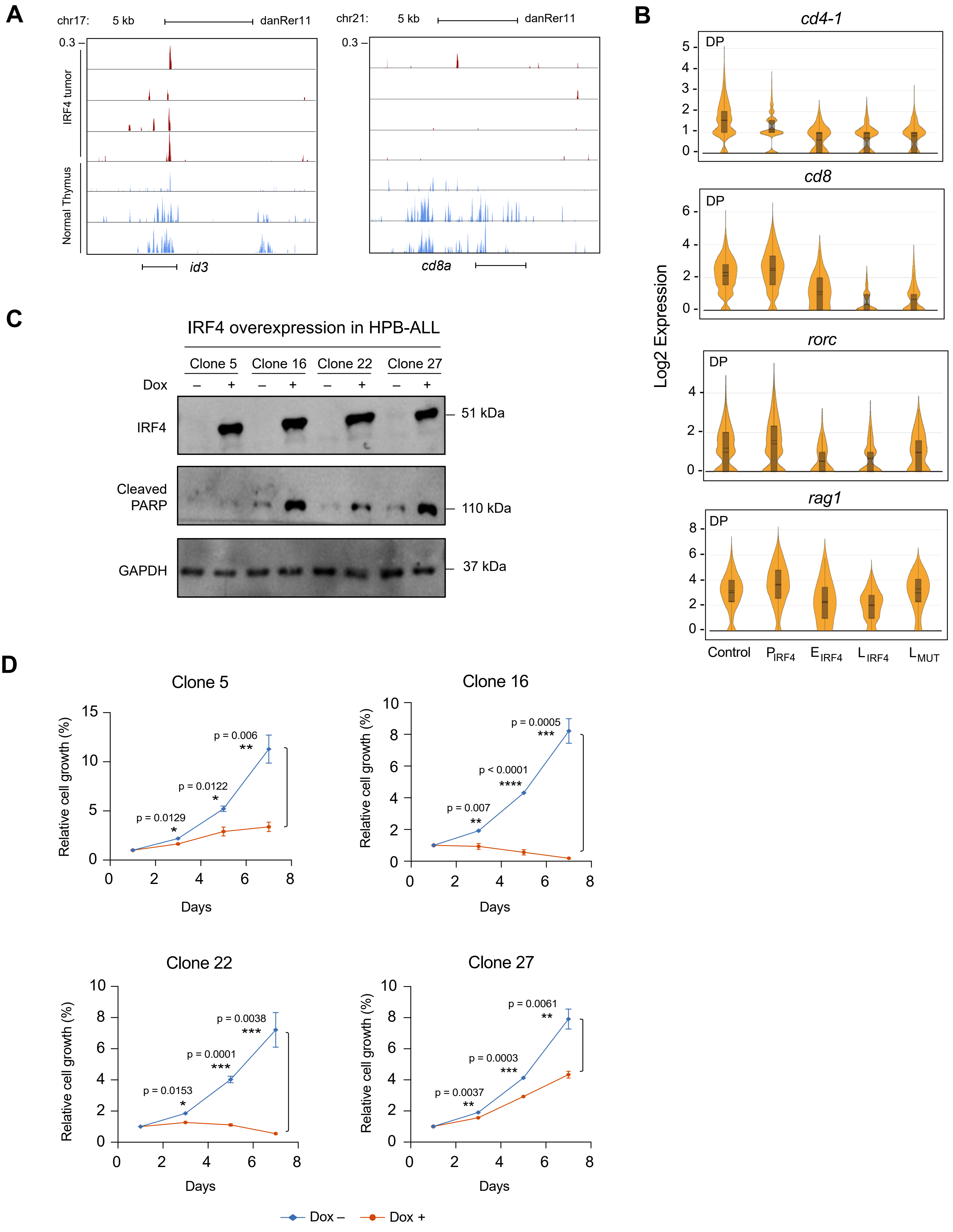

Supplementary Figure 6

Supplementary Fig. 6. Downregulation of p53 targets and *id3*. (A) ChIP-seq gene tracks representing H3K27ac signals at four gene loci in four zebrafish tumor samples and three normal thymus samples. **(B)** Violin plots showing the expression of *cd4-1*, *cd8a*, *rorc* and *rag1* in DP cell populations across tumor stages analyzed by scRNA-seq (total cell number after merging of biologically independent samples: Control n=1,042 cells; P_{IRF4} n=2,201 cells; E_{IRF4} n=66; L_{IRF4} n=473 cells; L_{MT} n=187 cells). See Fig.5C legend for the details of violin plots. **(C)** Western blot showing protein expression of IRF4 and cleaved PARP (apoptosis marker) in HPB-ALL cells after induction of IRF4 under the doxycycline (Dox)-inducible system. GAPDH was used as an internal control. **(D)** Cell growth after IRF4 overexpression by Dox treatment. n=3. Error bars (mean ± SEM). *p<0.05, **p<0.01, ***p<0.001, ****p<0.0001 by Student's two-tailed t-test, compared to Dox (-) cells. Source data are provided as a Source Data file.

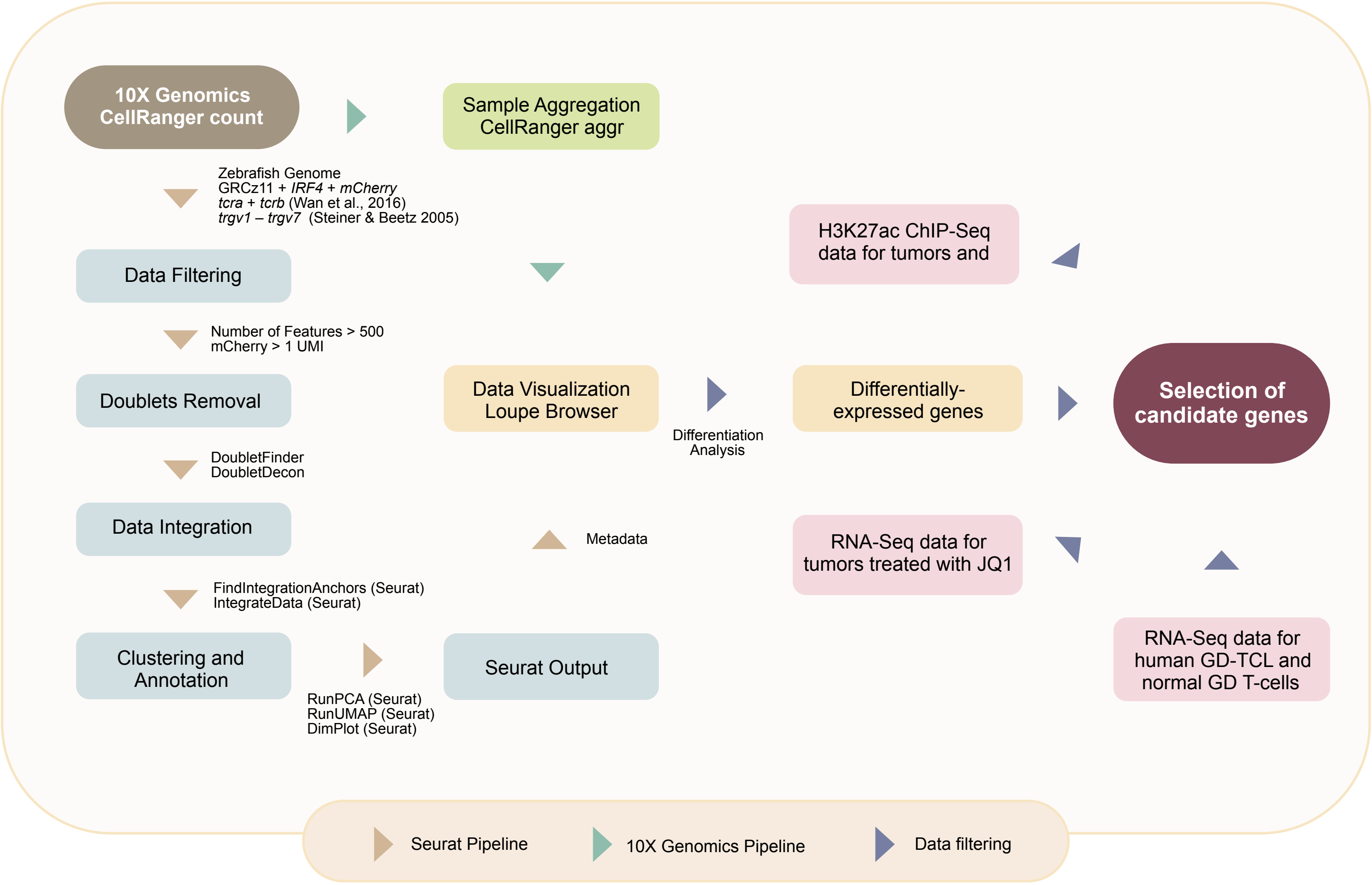

Supplementary Fig. 7 Flow of single cell sequencing analysis
