# Supplementary material for: IRF4 drives clonal evolution and lineage choice in a zebrafish model of T-cell lymphoma
Source: Nat Commun. 2022 May 3;13:2420. doi: 10.1038/s41467-022-30053-9 (PMC9065160; doi:10.1038/s41467-022-30053-9)
Supplement: Supplementary file 2 — Description of Additional Supplementary Files [file 41467_2022_30053_MOESM2_ESM.pdf]

Description of Additional Supplementary Files

File Name: Supplementary Data 1
Description: Marker genes in each cell population.

File Name: Supplementary Data 2
Description: Summary of differential binding analysis for H3K27ac ChIP-seq.

File Name: Supplementary Data 3
Description: Summary of super-enhancer analysis

File Name: Supplementary Data 4
Description: Summary of differential gene expression analysis for RNA-seq analysis JQ1 treatment.

File Name: Supplementary Data 5
Description: QC metrics for single-cell RNA-sequencing analysis
