# Supplementary material for: IRF4 drives clonal evolution and lineage choice in a zebrafish model of T-cell lymphoma
Source: Nat Commun. 2022 May 3;13:2420. doi: 10.1038/s41467-022-30053-9 (PMC9065160; doi:10.1038/s41467-022-30053-9)
Supplement: Supplementary file 8 — Reporting Summary [file 41467_2022_30053_MOESM8_ESM.pdf]

nature research

Corresponding author(s): Takaomi Sanda

Last updated by author(s): Mar 11, 2022

# Reporting Summary

Nature Research wishes to improve the reproducibility of the work that we publish. This form provides structure for consistency and transparency in reporting. For further information on Nature Research policies, see our Editorial Policies and the Editorial Policy Checklist.

## Statistics

For all statistical analyses, confirm that the following items are present in the figure legend, table legend, main text, or Methods section.

| n/a | Confirmed | |
|---|---|---|
| ☐ | ☒ | The exact sample size (*n*) for each experimental group/condition, given as a discrete number and unit of measurement |
| ☐ | ☒ | A statement on whether measurements were taken from distinct samples or whether the same sample was measured repeatedly |
| ☐ | ☒ | The statistical test(s) used AND whether they are one- or two-sided<br>*Only common tests should be described solely by name; describe more complex techniques in the Methods section.* |
| ☒ | ☐ | A description of all covariates tested |
| ☒ | ☐ | A description of any assumptions or corrections, such as tests of normality and adjustment for multiple comparisons |
| ☐ | ☒ | A full description of the statistical parameters including central tendency (e.g. means) or other basic estimates (e.g. regression coefficient) AND variation (e.g. standard deviation) or associated estimates of uncertainty (e.g. confidence intervals) |
| ☐ | ☒ | For null hypothesis testing, the test statistic (e.g. *F*, *t*, *r*) with confidence intervals, effect sizes, degrees of freedom and *P* value noted<br>*Give P values as exact values whenever suitable.* |
| ☒ | ☐ | For Bayesian analysis, information on the choice of priors and Markov chain Monte Carlo settings |
| ☒ | ☐ | For hierarchical and complex designs, identification of the appropriate level for tests and full reporting of outcomes |
| ☒ | ☐ | Estimates of effect sizes (e.g. Cohen's *d*, Pearson's *r*), indicating how they were calculated |

*Our web collection on statistics for biologists contains articles on many of the points above.*

## Software and code

Policy information about availability of computer code

| | |
|---|---|
| Data collection | Not applicable. |
| Data analysis | Microscopy image acquisition softwares and analyses - AxioVision software 3.0, Nikon NIS-Elements D and Olympus DP-BSW Ver.03.03<br><br>Fluorescence quantification - ImageJ 1.50i (NIH)<br><br>Flow cytometry analysis - BD FACSDiva<br><br>Statistics analysis - GraphPad Prism 9.3.1<br><br>Single-cell data analysis - Single-cell sequencing data were processed using the 10X Genomics software package CellRanger v3.1.0. The reads were aligned to the GRCz11/danRer11 genome, and mCherry, human IRF4 (ENSG00000137265), the TCR-α constant (trac) and β constant (trbc) genes reported in Wan et al.  were manually included in the annotation. Constructs generated using the consensus v-j-c sequence from zebrafish trgv1-trgv7 reported by Susann and Steiner in Nucleotide Database (NUCCORE) were also manually included. Zebrafish transcriptome data were generated by filtering the Ensembl annotations for protein-coding immunoglobulin and TCR genes that were defined in the GRCz11 GTF file. Individual read counts were performed using the CellRanger “count” function, which counted the reads based on the number of observed cell barcodes and generated a matrix for each cell and count. Then, all the samples (n=20) were combined into a single aggregated version to determine gene expression using both the CellRanger “aggr” function and Seurat R package v3.0 and were visualized using Loupe Browser v5.0 software.<br><br>Data integration - Doublet removal was performed by integrating the outputs of DoubletFinder and DoubletDecon. The R package Seurat v3.0 was used to filter, integrate and recluster the data. Each sample was filtered with gene features >500 and mCherrry expression ≥1 UMI. The anchors of each sample determined by assigning each anchors a score and construct an overall neighbor graph and compute their SNN graph |

using the FindIntegrationAnchors function (n=3,000). Later, the distance between datasets were defined by the number of anchors between datasets, the pairwise distance between datasets were compute and cluster to determine a guide tree. These integrated multiple datasets (n=20) into a single merge Seurat object by using the IntegrateData function. Principal component analysis (PCA) was performed using the RunPCA function with 20 dimensions (dims=20), followed by uniform manifold approximation and projection (UMAP) visualization of the PCA data using the RunUMAP function. The annotation were firstly done using the control fish scRNA-seq by manually select cells based on the marker gene expression. Then the same marker gene were applied to the rest of the individual tumor sample to annotate each cell cluster. In total there are 6 general cluster annotated (double negative DN, double positive DP, CD4 positive, CD8 positive, B-cells and Others). The single-cell clusters were visualized using the DimPlot function, and the gene expression plot was visualized using the FeaturePlot function. Cell populations were defined based on marker gene expression. All these functions are available in the Seurat R package v3.0. Violin plots, and gene expression images were generated using Loupe Browser v5.0. All TCR-g variable genes (trgv1-trgv7) were calculated using AddModuleScore by summing the manually constructed v-j-c counts and visualized using FeaturePlot. All these functions are available in Seurat R package v3.0.

Single-cell differential analysis- Significant marker genes were selected by comparing one cluster to all the other clusters using Loupe Browser v5.0 with the 'locally distinguishing' method to measure the significant genes in each cluster. Loupe Browser uses the sSeq methods for differential expression analysis which employs the negative binomial exact test. Differential expression data were retrieved from the average expression of each gene per sample and the heatmap plot using ComplexHeatmap R package version 2.1.0. p-values adjusted for multiple testing using the Benjamini-Hochberg procedure were generated by the Loupe Browser v5.0.

ChIP-seq analysis- ChIP-seq reads were mapped to zebrafish (Danio rerio GRCz11) reference genome using bowtie2 v2.4.1 program with default parameters. Aligned ChIP-seq reads were processed with samtools v1.12 rmdup package to remove duplicates. ChIP-Seq peaks were called with MACS2 software, version 2.1.1.2016030. Threshold for the braodpeaks called is P-value < 1E-3 and broadPeak cutoff is P-value < 1e-3 --broad-cutoff 1e-3 -p 1e-3 --broad --keep-dup 1 -f BAM --SPMR. After defined the broadPeaks, we use ROSE2 package version 1.1.0 to rank the enhancers into super-enhancers or typical enhancers by integrating zebrafish refseq ID into ROSE2 package, by stitching peaks within 12500 bps and exclude TSS of 2500 bps. Differential binding analysis were performed using DiffBind v3.0. The peaks of each sample were merge and consensus site between all samples (4 tumor vs 3 normal thymus) were compare using DESeq2 algorithm in DiffBind package. The significant region (FDR<0.05) reported by DiffBind were selected and group into Group I (log2FC >1) , Group II ( 1 < log2FC> -1 ) and Group III (log2FC<-1). 500bp from the center of each peakssignificant region (250bp to both ends) were extracted to perform motif enrichment analysis. Motif enrichment analysis using meme-chip function from MEME_Suite by utilizing the vertebrates PWN from Transfac and HOCOMOCO v11 motif database.

RNA-seq - Strand-specific library construction and sequencing of paired-end, 100-bp-long reads by the BGISeq500 were performed at the BGI Biotech Solutions (Hong Kong) Co Ltd (Hong Kong). RNA-seq datasets of DMSO-treated control samples and JQ1-treated samples were aligned to the GRCz11 danRer11 genome Ensembl annotation with ERCC spike-in information using STAR 2.5.2a with the parameter outFilterMultimapNmax set to 1. FeatureCount was used for the mapped reads in bam files to generate count tables based on the Ensembl gene annotation and ERCC annotation. Bioconductor package DESeq2 v1.12.4 was used to analyze differential gene expression using 3 DMSO vs 3 JQ1-treated samples.

For manuscripts utilizing custom algorithms or software that are central to the research but not yet described in published literature, software must be made available to editors and reviewers. We strongly encourage code deposition in a community repository (e.g. GitHub). See the Nature Research guidelines for submitting code & software for further information.

## Data

Policy information about availability of data

All manuscripts must include a data availability statement. This statement should provide the following information, where applicable:

- Accession codes, unique identifiers, or web links for publicly available datasets
- A list of figures that have associated raw data
- A description of any restrictions on data availability

The regular RNA-seq, ChIP-seq and single-cell RNA-seq data generated in this study have been deposited in the Gene Expression Omnibus (GEO) database under accession numbers "GSE139226 [https://www.ncbi.nlm.nih.gov/geo/query/acc.cgi?acc=GSE139226]" , "GSE166644[https://www.ncbi.nlm.nih.gov/geo/query/acc.cgi?acc=GSE166644]", "GSE166646[https://www.ncbi.nlm.nih.gov/geo/query/acc.cgi?acc=GSE166646]", "GSE184946[https://www.ncbi.nlm.nih.gov/geo/query/acc.cgi?acc=GSE184946]" and merge into a super-series and "GSE166650[https://www.ncbi.nlm.nih.gov/geo/query/acc.cgi?acc=GSE166646]", respectively.

The dataset for human GD T-cell lymphoma and normal GD T-cells has been reported and deposited under the dbGaP and GEO database under accession number "GSE107011[https://www.ncbi.nlm.nih.gov/geo/query/acc.cgi?acc=GSE107011]" and "phs001969[https://www.ncbi.nlm.nih.gov/projects/gap/cgi-bin/study.cgi?study_id=phs001969.v1.p1]". The dataset for Rag2-Myc zebrafish has been reported and deposited under GEO database under accession number "GSE108855 [https://www.ncbi.nlm.nih.gov/geo/query/acc.cgi?acc=GSE108855]"

Zebrafish genome and annotation version GRCz11 is available from Ensembl database.

# Field-specific reporting

Please select the one below that is the best fit for your research. If you are not sure, read the appropriate sections before making your selection.

☒ Life sciences ☐ Behavioural & social sciences ☐ Ecological, evolutionary & environmental sciences

For a reference copy of the document with all sections, see nature.com/documents/nr-reporting-summary-flat.pdf

# Life sciences study design

All studies must disclose on these points even when the disclosure is negative.

| | |
|---|---|
| Sample size | Sample sizes were chosen based on expected phenotypes, our previous experience and previous zebrafish studies.<br>1) Gutierrez A, et al. Pten mediates Myc oncogene dependence in a conditional zebrafish model of T cell acute lymphoblastic leukemia. J Exp Med 208, 1595-1603 (2011).<br>2) Mansour MR, et al. JDP2: An oncogenic bZIP transcription factor in T cell acute lymphoblastic leukemia. J Exp Med 215, 1929-1945 (2018).<br>3) Zhu et al. Activated ALK collaborates with MYCN in neuroblastoma pathogenesis. Cancer Cell. 2012 Mar 20;21(3):362-73<br><br>All the statistical analysis were done in GraphPad Prism software. A p-value less than 0.05 was considered statistically significant. The details of methods used can be found in each figure legend. |
| Data exclusions | No data was excluded in this study. |
| Replication | Tumor monitoring study was repeated using multiple independent animals for lck:IRF4 F0 (n=21) and F1 animals lck:IRF4;p53 wt/wt (n=79). Tumor monitoring study using multiple independent animals for all lines (lck:IRF4;p53 wt/wt, lck:IRF4;p53 wt/mut and lck:IRF4;p53 mut/mut and lck:mCherry) were replicated for at least two generations of offsprings. We observed similar findings.<br><br>Histology analyses were done in multiple biological replicates as follows - Tg(lck:mCherry;p53wt/wt) (n=8), Tg(lck:IRF4;p53wt/wt) (n=8) and Tg(lck:IRF4;p53wt/mut) (n=4). Histology for transplanted animals were done in 2 biological replicates for 3 different donors. We observed similar findings.<br><br>Single cell RNA-seq analysis were done with the following sample number: 1 control lck-mCherry, 5 preleukemic lck:IRF4, 3 early tumor lck:IRF4, 6 late tumor lck:IRF4, and 5 late tumor lck:IRF4;p53+/- fish.<br><br>Transplantation assay were replicated using 5 primary tumor fish donors. We observed similar findings.<br><br>Drug study was done in 5 control fish (treated with DMSO) and 5 fish (treated with JQ1). This study was replicated 2 times and we saw similar observation.<br><br>All qRT-PCR validations were done in technical duplicates from the following samples: control lck:mCherry n=1, lck:IRF4;p53 wt/wt n=9, lck:IRF4:p53 wt/mut n=6 and lck:IRF4:p53 mut/mut n=5.<br><br>Western blot analysis was done using 2 biological replicates.<br><br>Cell lines experiment was done using 2 biological replicates and 3 technical replicates. |
| Randomization | Tumor monitoring study using stable lines and downstream analyses were down using a specific founder animal. Off-springs share the same genetic background. Hence, there is no experiment that required randomization in this study. |
| Blinding | Tumor monitoring study using stable lines and downstream analyses were down using a specific founder animal. Off-springs share the same genetic background. Hence, there is no experiment that required blinding in this study. |

# Reporting for specific materials, systems and methods

We require information from authors about some types of materials, experimental systems and methods used in many studies. Here, indicate whether each material, system or method listed is relevant to your study. If you are not sure if a list item applies to your research, read the appropriate section before selecting a response.

## Materials & experimental systems

| n/a | Involved in the study | |
|---|---|---|
| ☐ | ☒ | Antibodies |
| ☐ | ☒ | Eukaryotic cell lines |
| ☒ | ☐ | Palaeontology and archaeology |
| ☐ | ☒ | Animals and other organisms |
| ☒ | ☐ | Human research participants |
| ☒ | ☐ | Clinical data |
| ☒ | ☐ | Dual use research of concern |

## Methods

| n/a | Involved in the study | |
|---|---|---|
| ☐ | ☒ | ChIP-seq |
| ☐ | ☒ | Flow cytometry |
| ☒ | ☐ | MRI-based neuroimaging |

## Antibodies

| | |
|---|---|
| Antibodies used | Anti-mouse IgG, HRP-linked Antibody (Cell Signaling Technology #7076)<br>Anti-mouse IgG, HRP-linked Antibody (Cell Signaling Technology #7076)<br>Anti-IRF4 antibody, (Santa Cruz, F-4, sc-48338)<br>Anti-Histone H3 (acetyl K27) antibody (Abcam, #ab4729) |

| | |
|---|---|
| | Cleaved PARP (Asp214) (D64E10) XP® Rabbit mAb (Cell Signaling Technology, #5625S)<br>Anti-GAPDH HRP Antibody (0411) (Santa Cruz, sc-47724) |
| Validation | Anti-mouse IgG was used as secondary antibody (dilution ration 1:10,000 in 5% milk 1x TBST)<br>Anti-rabbit IgG was used as secondary antibody (dilution ratio 1:10,000 in 5% milk 1x TBST)<br>Anti-IRF4 antibody was validated in Western Blot experiment. (dilution ratio 1:1000)<br>Anti-Histone H3 (acetyl K27) antibody was validated in ChIP-seq experiment. (1ug per reaction)<br>Cleaved PARP antibody was validated in Western Blot experiment. (dilution ratio 1:1000)<br>GAPDH-HRP was validated in Western blot experiment. (dilution ratio 1:1000)<br><br>These antibody for IRF4 have been validated by functional analyses in our previous studies.<br>1) Wong RWJ, et al. Enhancer profiling identifies critical cancer genes and characterizes cell identity in adult T-cell leukemia. Blood 130, 2326-2338 (2017).<br>2) Wong RWJ, et al. Feed-forward regulatory loop driven by IRF4 and NF-kappaB in adult T-cell leukemia/lymphoma. Blood 135, 934-947 (2020). |

# Eukaryotic cell lines

Policy information about cell lines

| | |
|---|---|
| Cell line source(s) | HPB-ALL was derived from human T-ALL patients and has been stocked in Sanda Laboratory. It was originally purchased from DSMZ cell bank. |
| Authentication | Authentication was done by DNA fingerprinting using the PowerPlex 1.2 System from Promega. |
| Mycoplasma contamination | Cell line was regularly tested for mycoplasma contamination to validate that they are negative. |
| Commonly misidentified lines<br>(See ICLAC register) | No commonly misidentified lines listed in the ICLAC database was used in this study. |

# Animals and other organisms

Policy information about studies involving animals; ARRIVE guidelines recommended for reporting animal research

| | |
|---|---|
| Laboratory animals | Four transgenic strains of zebrafish (Danio rerio) were established for this study (lck:IRF4, lck:IRF4;p53, lck:mCherry and rag2:IRF4)<br><br>Tumor monitoring study was done until the animals were 1 year old.<br><br>Single cell RNA-sequencing samples were harvested from 3 months old lck:mCherry (as control), 3 months old lck:IRF4 (with and without tumors), 10 months old lck:IRF4, and 9 months old (lck:IRF4;p53+/-).<br><br>Western blot analysis and regular RNA-seq samples were obtained from tumor cells of 10 months old lck:IRF4 zebrafish.<br><br>Immunocompromised rag2 E450fs mutant zebrafish strain was used for transplantation assay. Age of animals used were 3 months old.<br><br>Genders were not distinguished for these studies. |
| Wild animals | There is no wild animal used in this study. |
| Field-collected samples | There is no field-collected samples used in this study. |
| Ethics oversight | All zebrafish protocols (BR17-0351, R17-0353, BR18-1349, R18-1350, BR18-0526, R19-1182 BR21-0082, R21-0084) were approved by the Institutional Animal Care and Use Committee of the National University of Singapore and were performed according to their recommendations. |

Note that full information on the approval of the study protocol must also be provided in the manuscript.

# ChIP-seq

## Data deposition

☒ Confirm that both raw and final processed data have been deposited in a public database such as GEO.

☒ Confirm that you have deposited or provided access to graph files (e.g. BED files) for the called peaks.

| | |
|---|---|
| Data access links<br>*May remain private before publication.* | GEO Accession Token Description<br>GSE166644 yrwhimqytzondod H3K27ac ChIP-seq data |
| Files in database submission | GSM5076924 Tumor1_H3K27ac_ChIP-seq<br>GSM5076925 Tumor1_Input_ChIP-seq<br>GSM5602133 Tumor2_H3K27ac_ChIP-seq<br>GSM5602134 Tumor2_Input_ChIP-seq<br>GSM5602137 Tumor3_H3K27ac_ChIP-seq |

GSM5602138 Tumor3_Input_ChIP-seq
GSM5940346 Tumor4_H3K27ac_ChIP-seq
GSM5940347 Tumor4_Input_ChIP-seq
GSM5602139 Thymus2-H3K27ac_ChIP-Seq
GSM5602140 Thymus2-Input_ChIP-Seq
GSM5602141 Thymus3-H3K27ac_ChIP-Seq
GSM5602142 Thymus3-Input_ChIP-Seq
GSM5602143 Thymus5-H3K27ac_ChIP-Seq
GSM5602144 Thymus5-Input_ChIP-Seq

Genome browser session (e.g. UCSC)

https://genome.ucsc.edu/s/tbking/GSE166644%20H3K27ac%20ChIP%20GRCzv11%20Tumor%20vs%20Thymus

## Methodology

Replicates

For H3K27ac ChIP-seq, tumor cells were harvested from 4 lck:IRF4 zebrafish and 3 thymus from normal zebrafish. For each ChIP, sonicated chromatin was incubated with Dynabeads (Invitrogen) coated with H3K27ac (Abcam, #ab4729).

Sequencing depth

Single-end 50M reads

Antibodies

H3K27ac (Abcam, #ab4729)

Peak calling parameters

-p 1e-3 --broad-cutoff 1e-3 --broad --keep-dup 1 -f BAM --SPMR

Data quality

more than 20k broadPeaks reported in each H3K27ac ChIP

Software

macs2 version 2.1.1.2016030

# Flow Cytometry

## Plots

Confirm that:

☒ The axis labels state the marker and fluorochrome used (e.g. CD4-FITC).

☒ The axis scales are clearly visible. Include numbers along axes only for bottom left plot of group (a 'group' is an analysis of identical markers).

☒ All plots are contour plots with outliers or pseudocolor plots.

☐ A numerical value for number of cells or percentage (with statistics) is provided.

## Methodology

Sample preparation

Cells were harvested from kidney marrow of wild-type zebrafish and from whole body lck:IRF4;p53+/+ zebrafish. Cells were resuspended and kept in sample preparation buffer (PBS with 20% FBS) during flow cytometry analysis.

Instrument

BD FACSAria II

Software

BD FACSDiva

Cell population abundance

Cell population was analyzed by gating strategy outlined below. .

Gating strategy

For the analysis of different hematopoietic cell population, cells were prepared from wild-type adult zebrafish kidney and mCherry-sorted cells from the Tg(lck:IRF4;p53wt/wt) fish. Cells were gated on FSC-H and FSC-A plot to eliminate doublets and then on FSC versus SSC plot to obtain hematopoietic lineage profiles for erythrocytes, granulocytes and monocytes, lymphocytes, and blood cell precursors.

For sorting of mCherry-positive cells from transgenic lines, cells were gated on FSC-H and FSC-A plot to eliminate doublets and then on FSC versus SSC plot and then top 50-80% mCherry-positive cells were gated and sorted.

☒ Tick this box to confirm that a figure exemplifying the gating strategy is provided in the Supplementary Information.
